# Supplementary material for: Determining the pneumococcal conjugate vaccine coverage required for indirect protection against vaccine-type pneumococcal carriage in low and middle-income countries: a protocol for a prospective observational study
Source: BMJ Open. 2018 May 18;8(5):e021512. doi: 10.1136/bmjopen-2018-021512 (PMC5961565; doi:10.1136/bmjopen-2018-021512)
Supplement: Author's manuscript [file bmjopen-2018-021512.draft_revisions.pdf]

BMJ Open

BMJ Open is committed to open peer review. As part of this commitment we make the peer review history of every article we publish publicly available.

When an article is published we post the peer reviewers' comments and the authors' responses online. We also post the versions of the paper that were used during peer review. These are the versions that the peer review comments apply to.

The versions of the paper that follow are the versions that were submitted during the peer review process. They are not the versions of record or the final published versions. They should not be cited or distributed as the published version of this manuscript.

BMJ Open is an open access journal and the full, final, typeset and author-corrected version of record of the manuscript is available on our site with no access controls, subscription charges or pay-per-view fees (http://bmjopen.bmj.com).

If you have any questions on BMJ Open's open peer review process please email info.bmjopen@bmj.com

BMJ Open

Determining the pneumococcal conjugate vaccine coverage required for indirect immunity in low- and middle-income countries: a protocol for a prospective observational study

Journal:	*BMJ Open*
Manuscript ID	bmjopen-2018-021512
Article Type:	Protocol
Date Submitted by the Author:	09-Jan-2018

Complete List of Authors:	Chan, Jocelyn; Murdoch Childrens Research Institute, Pneumococcal Research Group; The University of Melbourne, Department of Paediatrics<br>Nguyen, Cattram; Murdoch Childrens Research Institute, Pneumococcal Research; The University of Melbourne, Department of Paediatrics<br>Lai, Jana; Murdoch Childrens Research Institute, Pneumococcal Research Group<br>Dunne, Eileen; Murdoch Childrens Research Institute, Pneumococcal Research Group; The University of Melbourne, Department of Paediatrics<br>Andrews, Ross; Menzies School of Health Research, Charles Darwin University, Global & Tropical Health Division; Australian National University, National Centre for Epidemiology & Population Health<br>Blyth, Christopher C.; University of Western Australia, School of Medicine; Princess Margaret Hospital, Department of Infectious Diseases<br>Datta, Siddhartha; World Health Organization<br>Fox, Kim; World Health Organization, Regional Office for the Western Pacific<br>Ford, Rebecca ; Papua New Guinea Institute of Medical Research<br>Hinds, Jason; St George's- University of London, Institute for Infection and Immunity; London Bioscience Innovation Centre, BUGS Bioscience<br>La Vincente, Sophie; Murdoch Childrens Research Institute, Pneumococcal Research Group<br>Lehmann, Deborah; Telethon Kids Institute, University of Western Australia, Wesfarmers Centre for Vaccines and Infectious Diseases<br>Lim, Ruth; Murdoch Childrens Research Institute, Pneumococcal Research Group<br>Mungun, Tuya ; Ministry of Health, National Center of Communicable Diseases (NCCD)<br>Newton, Paul ; Lao-Oxford-Mahosot Hospital-Wellcome Trust Research Unit , Microbiology Laboratory, Mahosot Hospital; University of Oxford, Centre for Tropical Medicine and Global Health<br>Phetsouvanh, Rattanaphone; Lao-Oxford-Mahosot Hospital-Wellcome Trust Research Unit, Microbiology Laboratory, Mahosot Hospital; University of Oxford, Centre for Tropical Medicine and Global Health<br>Pomat, Willie; PNG Institute of Medical Research, Infection and Immunity; Telethon Kids Institute, University of Western Australia, Wesfarmers Centre for Vaccines and Infectious Diseases<br>Xeuatvongsa, Anonh; Ministry of Health, National Immunization Programme<br>von Mollendorf, Claire; Murdoch Childrens Research Institute, Pneumococcal Research Group; The University of Melbourne, Department

of Paediatrics
Dance, David; Lao-Oxford-Mahosot Hospital-Wellcome Trust Research Unit, Microbiology Laboratory, Mahosot Hospital; London School of Hygiene and Tropical Medicine, Faculty of Infectious and Tropical Diseases
Satzke, Catherine; Murdoch Childrens Research Institute, Pneumococcal Research; The University of Melbourne at the Peter Doherty Institute for Infection and Immunity, Department of Microbiology and Immunology
Muholland , Kim; Murdoch Childrens Research Institute, Pneumococcal Research Group; London School of Hygiene and Tropical Medicine, Department of Infectious Disease Epidemiology
Russell, F. M.; Murdoch Childrens Research Institute, Pneumococcal Research Group; The University of Melbourne, Centre for International Child Health, Department of Paediatrics

Keywords: Public health < INFECTIOUS DISEASES, Paediatric infectious disease & immunisation < PAEDIATRICS, Respiratory infections < THORACIC MEDICINE

SCHOLARONE™ Manuscripts

For peer review only

DETERMINING THE PNEUMOCOCCAL CONJUGATE VACCINE COVERAGE REQUIRED FOR INDIRECT IMMUNITY IN LOW- AND MIDDLE-INCOME COUNTRIES: A PROTOCOL FOR A PROSPECTIVE OBSERVATIONAL STUDY

Authors:

Chan J[1,2], Nguyen CD[1,2], Lai JYR[1], Dunne EM[1,2], Andrews R[3,4], Blyth CC[5,6], Datta S[7], Fox K[8], Ford R[9], Hinds J[10,11], La Vincente S[1] , Lehmann D[12], Lim R[1], Mungun T[13], Newton PN[14,15], Phetsouvanh R[14,15*], Pomat W[9,12], Xeuatvongsa A[16], von Mollendorf C[1,2], Dance DAB[14,17], Satzke C[1,18], Mulholland K[1,19], Russell FM[1,20] for the PneuCAPTIVE Protocol Group

1. Pneumococcal Research Group, Murdoch Children's Research Institute, Melbourne, Australia.
2. Department of Paediatrics, The University of Melbourne, Melbourne, Australia.
3. Global & Tropical Health Division, Menzies School of Health Research, Charles Darwin University, Darwin, Australia.
4. National Centre for Epidemiology & Population Health, Australian National University, Canberra, Australia
5. University of Western Australia, School of Medicine, Perth, Australia;
6. Department of Infectious Diseases, Princess Margaret Hospital, Perth, Australia;
7. World Health Organization, Vientiane, Lao People's Democratic Republic
8. Regional Office for the Western Pacific, World Health Organization, Manila, Philippines.
9. Papua New Guinea Institute of Medical Research, Infection and Immunity Unit, Goroka, Papua New Guinea.
10. Institute for Infection and Immunity, St George's- University of London, London, United Kingdom.
11. BUGS Bioscience, London Bioscience Innovation Centre, London, United Kingdom.
12. Wesfarmers Centre for Vaccines and Infectious Diseases, Telethon Kids Institute, University of Western Australia, Perth, Australia.
13. National Center of Communicable Diseases (NCCD), Ministry of Health, Ulaanbaatar, Mongolia.
14. Lao-Oxford-Mahosot Hospital-Wellcome Trust Research Unit (LOMHWRU), Microbiology Laboratory, Mahosot Hospital, Vientiane, Lao PDR.
15. Centre for Tropical Medicine and Global Health, University of Oxford, Oxford, United Kingdom.
16. National Immunization Programme, Ministry of Health, Vientiane, Lao PDR.
17. Faculty of Infectious and Tropical Diseases, London School of Hygiene and Tropical Medicine, London, United Kingdom
18. Department of Microbiology and Immunology, The University of Melbourne at the Peter Doherty Institute for Infection and Immunity, Melbourne, Australia

For peer review only

19. Department of Infectious Disease Epidemiology, London School of Hygiene and Tropical Medicine, London, United Kingdom.
20. Centre for International Child Health, Department of Paediatrics, The University of Melbourne, Melbourne, Australia.

*Deceased

Corresponding author: Jocelyn Chan

Pneumococcal Research Group,
Murdoch Children's Research Institute
50 Flemington Road, Parkville 3052 VIC
Australia

Email: jocelyn.chan@mcri.edu.au

Telephone: +61 3 9345 4968

For peer review only

ABSTRACT

Introduction

Pneumococcal conjugate vaccines (PCVs) prevent disease through both direct protection of vaccinated individuals, and indirect protection of unvaccinated individuals by reducing nasopharyngeal (NP) carriage and transmission of vaccine-type pneumococci. While the indirect effects of PCV vaccination are well described, the PCV coverage required to achieve the indirect effects is unknown. We will investigate this using hospital-based NP pneumococcal carriage surveillance at three sites in the Asia-Pacific region.

Methods and analysis

We are recruiting children aged 2-59 months admitted to participating hospitals with acute respiratory infection in Lao People's Democratic Republic, Mongolia and Papua New Guinea. Thirteen-valent PCV (PCV13) status is obtained from written records. NP swabs are collected according to standard methods, screened using *lytA* qPCR and serotyped by microarray. Village-level vaccination coverage, for the resident communities of the recruited cases, is determined using administrative data or community survey. Our analysis will investigate the relationship between prevalence of PCV13 carriage among under-vaccinated cases (indirect effects) and vaccine coverage using generalised estimating equations.

Ethics and dissemination

Ethical approval has been obtained from the relevant ethics committees at participating sites. The results are intended for publication in open-access peer-reviewed journals and will demonstrate methods suitable for low- and middle-income countries to monitor vaccine impact, and inform vaccine policy makers about the PCV coverage required to maximise the effects of PCV.

STRENGTHS AND LIMITATIONS

- This protocol describes a method for monitoring the indirect effects of pneumococcal conjugate vaccines (PVCs) on pneumococcal carriage in low- and middle-income countries, in the absence of baseline pre-PCV data
- The use of molecular serotyping microarray enables detection of multiple serotype carriage and serotype-specific carriage density
- The inclusion of three sites, which have contrasting vaccine schedules and pneumococcal epidemiology, will enable us to explore factors which may modify the vaccine coverage required to achieve indirect effects however variations in methods and settings may impact on the comparability of our results across the three sites

For peer review only

INTRODUCTION

Infections due to *Streptococcus pneumoniae* (the pneumococcus), including pneumonia, meningitis and septicaemia, are a leading cause of morbidity and mortality among children and elderly, especially in low- and middle-income countries.[1] The main reservoir for the pneumococcus is the human nasopharynx. Pneumococcal carriage peaks in young children, an important group for transmission of pneumococci to older age groups,[2 3] and is a precursor for disease.[4] There are over 90 known serotypes of pneumococci, with differing capacities for causing disease.[5]

The introduction of the pneumococcal conjugate vaccine (PCV) has resulted in substantial reductions in pneumococcal disease in many settings.[6] These reductions are mediated by direct effects on vaccinated children as well as indirect effects on under-vaccinated children and adults through a reduction in transmission and subsequent nasopharyngeal (NP) carriage of pneumococcal serotypes included in the vaccine.[7-10] The indirect effects account for a substantial component of the overall vaccine effect. Following the introduction of the 7-valent PCV (PCV7) into the routine vaccination program in the USA, twice as many invasive cases were prevented through indirect effects compared to direct effects.[11]

The magnitude of indirect effects following introduction of PCV varies considerably by setting. Reductions in invasive pneumococcal disease (IPD) among adults ranged from 8.8% among adults in Denmark three years after PCV implementation, to 70% reduction among adults in Taiwan, seven years after PCV implementation.[12] A review of the literature found that higher rates of coverage, higher baseline rates of pneumococcal disease and greater time elapsed since PCV introduction were all associated with greater degrees of indirect effects.[12] However, the majority of these studies were conducted in high-income country settings using a similar vaccine schedule, with relatively high levels of vaccine coverage.

The threshold of vaccination coverage required to achieve significant indirect PCV effects is not well understood. Studies of NP carriage indicate that there is variability in the coverage required to achieve significant indirect effects. Two studies in the US examined this question for the 13-valent PCV (PCV13) that superseded 7-valent (PCV7) immunisation. A vaccine coverage of 58% among American Indian children in south-western USA and 75% coverage among children in Massachusetts resulted in a 50% decline in the prevalence of carriage of six PCV13 serotypes (i.e. PCV13 types not included in PCV7) carried by under-vaccinated children.[13 14] The US uses a 3+1 schedule (three primary doses with a booster) and had a catch-up program up to five years of age. Many low- and middle-income countries utilise a 3+0 schedule. [15] More data are needed from a range of low- and middle-income countries to determine the vaccination coverage required to maximise the indirect effects of PCVs, and to determine factors which may modify the vaccine coverage required for indirect effects, such as baseline carriage prevalence, indicating intensity of transmission, vaccine schedules and use of catch-up campaigns.

Existing systematic reviews indicate that vaccine schedules do not impact on the degree of indirect effects on IPD, however the studies included in these reviews predominantly use a 3+1 schedule or a

For peer review only

2+1 schedule (two primary doses with a booster) and have limited studies using PCV13.[12 16] Few studies using the 3+0 schedule (three primary doses with no booster) are available in the published literature, despite the 3+0 schedule being widely used, especially in low- and middle-income countries.

Despite the gradual introduction of PCVs in low- and middle-income countries over the last decade, there have been few studies published on the impacts of PCV in these settings.[17 18] This is because the predominant method for assessing vaccine impact, IPD surveillance, is costly and resource-intensive to establish – requiring laboratory capacity as well as the collection of large numbers of samples, obtained using aseptic techniques, in order to detect a relatively rare outcome.[19] Furthermore, baseline data prior to vaccine introduction required for impact evaluations are often not available in these settings.

In the absence of IPD surveillance, low- and middle-income countries require a method to evaluate direct and indirect vaccine effects. We propose using NP carriage surveillance in children hospitalised with acute respiratory infection to monitor the indirect effects of PCV13 as part of the PneuCAPTIVE study (PNEUmococcal CArriage in Pneumonia To Investigate Vaccine Effects).

In this context, we refer to “carriage” as the detection of pneumococci in the nasopharynx of a child with acute respiratory infection. A reduction in vaccine-type (VT) pneumococcal carriage will likely reflect reductions in disease due to VT pneumococci, since carriage is a precursor to disease.[4] Existing studies among healthy children have demonstrated reductions in VT carriage following PCV introduction, while overall carriage remains stable due to replacement with non-vaccine type (NVT) carriage.[20 21]

We aim to determine the PCV13 coverage required to demonstrate substantial indirect effects of PCV using NP carriage surveillance in children hospitalised with acute respiratory infection (ARI) in three settings within the Asia-Pacific region. We are focussing on children under five years of age because studies have shown that children are the main reservoir for pneumococci, and reductions in transmission within this age group are likely to result in reductions in VT carriage among older age groups. [2 3 22]

As this is a novel method for determining indirect effects, we will also aim to determine whether changes in patterns of VT and NVT pneumococcal carriage among children with ARI are reflective of changes in serotypes circulating in the community, noting that carriage in our cohort may be more reflective of serotypes causing disease. [5 23]

OBJECTIVES

Our objectives are to: (1) investigate the relationship between PCV13 coverage and VT carriage among under-vaccinated children (indirect effects) aged 2-59 months with an ARI in Lao People’s Democratic Republic (Lao PDR), Mongolia and Papua New Guinea (PNG); (2) describe monthly trends in VT carriage prevalence among cases and contacts; (3) investigate the relationship between PCV13 coverage and VT carriage among under-vaccinated children (indirect effects) living in the

For peer review only

community; and (4) compare the PCV13 coverage required to demonstrate indirect effects of PCV13 by site and determine the degree to which site-specific factors, such as baseline pneumococcal carriage rates and densities, vaccine schedule and use of catch-up campaigns, account for differences in the PCV13 coverage required to demonstrate indirect effects.

METHODS

Study design

We are conducting prospective hospital-based observational studies in Lao PDR, Mongolia and PNG. We are recruiting children 2-59 months of age presenting with ARI and obtaining NP swabs to determine prevalence and density of VT carriage. We are determining the PCV13 status of each case using written record. Recruitment will occur over at least three years post-PCV13 introduction at each site.

Concurrently, we are determining vaccination coverage at the resident village or subdistrict of each recruited case, using either administrative data or vaccination coverage surveys.

Study settings

Lao PDR site

The Lao PDR PneuCAPTIVE study is embedded within a hospital-based study of ARI aetiology, in collaboration with Lao Oxford Mahosot Hospital-Wellcome Trust Research Unit (LOMWRU), the World Health Organization (WHO) and the Lao PDR Ministry of Health [24]. PCV13 was introduced in October 2013, using a 3+0 schedule at 6, 10 and 14 weeks of age and a catch-up program up to 12 months of age (The PNG PneuCAPTIVE study represents a collaboration between the PNG Institute of Medical Research (IMR), Telethon Kids Institute, the University of Western Australia and MCRI. It is an extension of a pneumonia aetiology study that commenced in 2013 [25]. PCV13 was introduced to PNG in October 2014 using a 3+0 schedule at 1, 2 and 3 months of age (Table 1), however was not widely distributed in the Eastern Highlands Province until late 2015. We are recruiting cases at the Eastern Highlands Provincial Hospital, the major referral hospital for the Eastern Highland Province, as well as nearby clinics in Goroka, the capital of the Eastern Highlands Province. In PNG, we are also recruiting caregivers, as well as community contacts, defined as children 0-59 months of age, who have slept in the same house as or played with the case during the preceding three weeks. This will enable us to determine whether changes in patterns of VT pneumococcal carriage in the hospitalised cases are reflective of changes within the community, as well as to examine indirect effects in the adult age group.

Table 1). We will recruit cases at Mahosot Hospital, one of the largest paediatric referral hospitals in Vientiane, the capital of Lao PDR.

Mongolia site

The Mongolian PneuCAPTIVE study is embedded within a hospital-based vaccine impact evaluation, which is conducted in partnership between Murdoch Children's Research Institute (MCRI), WHO and the Mongolian Ministry of Health. PCV13 was introduced in June 2016, using a modified 2+1 schedule at 2, 4 and 9 months of age, within the two 'Phase 1' districts within Ulaanbaatar, the capital of

For peer review only

Mongolia as part of a phased introduction (Table 1), with a catch-up program of two doses one month apart for those up to 24 months of age. We are recruiting cases, residing in the two phase 1 districts, at the two district hospitals and the tertiary referral paediatric hospital for Mongolia, the Maternal and Child Hospital (MCH).

PNG site

The PNG PneuCAPTIVE study represents a collaboration between the PNG Institute of Medical Research (IMR), Telethon Kids Institute, the University of Western Australia and MCRI. It is an extension of a pneumonia aetiology study that commenced in 2013 [25]. PCV13 was introduced to PNG in October 2014 using a 3+0 schedule at 1, 2 and 3 months of age (Table 1), however was not widely distributed in the Eastern Highlands Province until late 2015. We are recruiting cases at the Eastern Highlands Provincial Hospital, the major referral hospital for the Eastern Highland Province, as well as nearby clinics in Goroka, the capital of the Eastern Highlands Province. In PNG, we are also recruiting caregivers, as well as community contacts, defined as children 0-59 months of age, who have slept in the same house as or played with the case during the preceding three weeks. This will enable us to determine whether changes in patterns of VT pneumococcal carriage in the hospitalised cases are reflective of changes within the community, as well as to examine indirect effects in the adult age group.

Table 1: Key aspects of the 13-valent pneumococcal conjugate vaccination (PCV13) program by site, Lao People's Democratic Republic (PDR), Mongolia and Papua New Guinea (PNG)

	Lao PDR	Mongolia	PNG
Year of PCV13 introduction	Oct 2013	Jun 2016	Oct 2014
Location of PCV13 introduction	National	Two districts in Ulaanbaatar	National
PCV13 schedule	3+0 (6,10 and 14 weeks)	2+1 (2,4, and 9 months)	3+0 (1, 2 and 3 months)
Presence of a catch-up program	Catch-up of three doses up to 12 months of age	Catch-up of two doses two months apart up to 24 months of age	None

Case recruitment and data collection

Participant recruitment and data collection are consistent across the three sites; however there are some local adaptations to the protocol at each site which are summarised in Table 2. These adaptations are due to the PneuCAPTIVE study being nested within other existing studies, described above.

Cases are eligible for inclusion in the PneuCAPTIVE study if they are 2-59 months of age, and presenting with ARI (defined in Table 2 below). All cases with fever or respiratory symptoms are screened for inclusion. In Mongolia, we are restricting recruitment to patients living within the two

For peer review only

'Phase 1' districts which have commenced PCV13 in 2016. In PNG, we are restricting recruitment to patients living within one hour of the town as follow-up is logistically challenging.

Regarding recruitment, in Lao PDR, study staff are screening potential recruits from Monday to Friday each week. However, they are obtaining clinical information from medical records for all eligible cases, including those admitted at weekends to ensure we have a representative sample. In Mongolia, caregivers of all eligible children presenting to the hospital will be approached for recruitment as part of the larger PCV impact study. However, for the purposes of this study we will select a random sample of 33 cases per month for microbiological testing. In PNG recruitment takes place 4-5 days per week.

Table 2: Patient eligibility by site, Lao People's Democratic Republic (PDR), Mongolia and Papua New Guinea (PNG)

**Site**	Lao PDR	Mongolia	PNG
**Inclusion criteria**	2-59 months of age and presenting with:		
	Fever (history or measured) AND one of cough OR dyspnoea OR rhinitis OR abnormal chest auscultation	Cough OR dyspnoea AND tachypnoea* OR hypoxia OR chest indrawing	Cough AND tachypnoea* AND lower chest wall indrawing
**Exclusion criteria**	-	Lives outside Phase 1 district<br>Admitted with pneumonia in the last 14 days	Lives ≥ 1 hour outside town OR hospitalisation in past 14 days
**Recruitment site**	Inpatient setting only	Inpatient setting only	Inpatient and outpatient setting
**Sampling**	Monday-Friday	Random (33 per month)	Convenience (Monday-Friday)

* Tachypnoea is defined as greater than or equal to 50 breaths per minute

After determining eligibility and obtaining informed parental consent, we complete a questionnaire to obtain: demographic data, clinical data, PCV13 status, and risk factors for vaccination and NP carriage, including prior antibiotic use (see analysis section below for complete list). Vaccination status is determined using written records – either parent-held immunisation records or health centre administrative records.

We then collect an NP swab according to WHO guidelines and store it in 1 ml skim milk tryptone glucose glycerol (STGG) medium. [26] Swabs are vortexed, aliquoted, and stored frozen at -80^{0}C within 8 hours of collection, and transported to the Pneumococcal Research laboratory at MCRI on dry ice or in liquid nitrogen, where they will be stored at -80^{0}C.

Laboratory methods

All samples are screened for the presence of pneumococci using real-time quantitative PCR (qPCR) assay targeting the pneumococcal *lytA* gene.[27] Genomic DNA are extracted from 100 µl of STGG using a MagNA Pure LC Machine (Roche) using the DNA Isolation Kit III (Bacteria, Fungi) (Roche) the following an enzymatic lysis treatment. The pneumococcal load is estimated by reference to a standard curve.

All swabs that are *lytA* positive or equivocal are molecular serotyped using BµG@S Senti-SP v1.5 microarray (BUGS Bioscience) as previously described. [28]

Serotype-specific pneumococcal density is calculated using the relative abundance of each serotype identified, as determined using microarray and interpreted with the assistance of a Bayesian random effects model as previously described,[28 29] and the overall pneumococcal load as determined by the *lytA* qPCR.

Key definitions

The primary outcome, VT carriage, is defined as the NP carriage of at least one pneumococcal serotype included in the PCV13 vaccine, i.e. serotypes 1, 3, 4, 5, 6A, 6B, 7F, 9V, 14, 18C, 19A, 19F, and 23F. A secondary outcome is VT carriage density (CFU/mL) which is defined as an aggregate of the serotype-specific density for each of the VTs carried by the case, and will be reported as a continuous variable using a log scale to account for large variations in density and a skewed distribution.

A case will be defined as 'vaccinated' if they have documented evidence of receiving an adequate number of PCV doses to provide a protective immune response against vaccine serotypes at least 14 days prior to study enrolment.[30] For children < 12 months of age, an adequate number of PCV doses is defined as two or more PCV13 doses. For children 12 months of age or older, an adequate number of doses is defined as receipt of 2 doses in the first year of life or at least 1 dose after the age of 12 months. Conversely, a case will be defined as 'under-vaccinated' if they have received less than the adequate number of PCV doses. Sensitivity analyses will be conducted using varying definitions of vaccinated including receiving at least one dose of vaccine at any age.

Vaccine coverage data collection

For each under-vaccinated case recruited, we are determining their resident village or subdistrict vaccination coverage. In Mongolia and Lao PDR, we are using administrative data, whereas in PNG, we are conducting community surveys within 10 days of discharge in the village where the case is living (Table 3). In Mongolia, we have validated the newly introduced electronic immunisation record against clinic health records, finding a high degree of concordance.[31] We are also in the process of validating the population registers at health centres in Mongolia.

Table 3: Vaccination coverage data by site, Lao People's Democratic Republic (PDR), Mongolia and Papua New Guinea

**Site**	Lao PDR	Mongolia	Papua New Guinea

For peer review only

**Source of numerator data**	Health centre records	Electronic immunisation record	Community surveys
**Source of denominator data**	Lao PDR Population and Housing Census 2015	Health centre population register*	

* All children are required to be registered at the health centre servicing their resident subdistrict in order to receive health services.

Data management

In Lao PDR and Mongolia, study staff are double-entering data using a RedCAP (Research Electronic Data Capture) and Microsoft Access (Microsoft Corporation) database, respectively. In PNG, data are checked by a monitor prior to being entered into Filemaker Pro (FileMaker Inc.). We are conducting regular double-entry discrepancy checks and logic checks using Stata Statistical Software (College Station, TX: StataCorp LLC).

Analysis

All analyses will be completed using Stata Statistical Software (College Station, TX: StataCorp LLC). We will summarise continuous variables using mean and standard deviation (or median and interquartile range for non-symmetrical data). Categorical variables will be summarised using frequency counts and percentages.

Objective 1: Relationship between vaccine coverage and indirect effects

We want to investigate the relationship between prevalence and density of VT carriage among under-vaccinated cases (indirect effects) and subdistrict/village PCV coverage. We will use an adaptation of a method used to estimate indirect protection for an oral cholera vaccine which exploits heterogeneities in vaccine coverage at the subdistrict/village level, comparing prevalence and density of VT carriage among under-vaccinated children from subdistricts/villages with differing levels of vaccine coverage.[32 33]

This will be done using multivariable models with VT carriage prevalence or density in ARI cases as the outcome variable and PCV coverage at the child's place of residence at the time of admission as the exposure variable. We will use generalised estimating equations to account for clustering at the subdistrict/village level.

To identify confounders for adjustment, we will constructed directed acyclic graphs (DAG). DAGs include all variables potentially related to exposure and outcome, connected using uni-directional arrows showing causal relationships between variables. The graph identifies potentially confounding pathways and allows investigators to determine variables that should be controlled for to obtain unbiased effect estimates. As there are likely to be unique confounders between sites, we will develop site-specific DAGs. We will use DAGitty.net (version 2.3) software to identify minimally sufficient confounding subsets for adjustment.

For peer review only

For each site, we will construct a similar model using overall pneumococcal carriage as the dependent variable. This model will act as a bias indicator since PCV coverage is not expected to affect levels of overall pneumococcal carriage due to replacement carriage with non-VTs (NVTs), although complete replacement to baseline levels can take several years.[20 34 35] Therefore, we will restrict this analysis to the latter part of the study period, when descriptive analyses indicate the replacement is complete.

Objective 2: VT carriage prevalence among cases and contacts, by calendar month (PNG only)
Crude and adjusted monthly VT carriage prevalence will be estimated within rolling seven-month intervals. This will be done separately for cases and contacts. To account for differences in age between cases and community contacts, the carriage prevalence will be adjusted using direct standardisation (standardised to the case population over the entire study period). We are using rolling seven-month intervals to present smooth curves, and to assess trends over time.

Objective 3: Relationship between vaccine coverage and indirect effects among community contacts (PNG only)
To investigate whether relationship between vaccine coverage and indirect effects as observed among cases with ARI are reflective of the relationship between vaccine coverage and indirect effects in the wider community, we will apply the same model described above to under-vaccinated community contacts. We will construct multivariable models with VT carriage prevalence or density among under-vaccinated community contacts as the outcome variable and PCV coverage at the child's place of residence at the time of admission as the exposure variable. We will be using the same vaccine coverage data as for the cases.

Objective 4: Comparison of vaccine coverage required for indirect effects across sites
We will compare differences in the PCV13 coverage required to demonstrate indirect effects of PCV13 qualitatively, by site and in relation to vaccine schedule and use of catch-up campaigns. Inferential statistics are unlikely to be suitable with the inclusion of only three sites and comparability between sites is limited due to variations between them.

Power calculation
Power calculations were performed using nQuery Advisor + nTerim 4.0. Calculations were based on sample size methods for logistic regression models with a continuous covariate (i.e. PCV coverage) and additional covariates, with inflation to account for clustering within villages. Power calculations assumed VT carriage prevalence of 20% in Lao PDR and Mongolia, and 40% in PNG at the mean PCV coverage level,[36] and VT carriage prevalence of 10% in Lao PDR and Mongolia and 30% in PNG at one standard deviation above the mean PCV coverage level. Assuming a significance level of 0.05, allowing for adjustment using multiple covariates with an r-squared of 0.4, and that 50% of the cases are under-vaccinated, a sample size of 1200 cases per site would provide between 99% power to determine the proportion of cases carrying VT pneumococcus at varying levels of village vaccine coverage. The power calculation has been adjusted to account for clustering by village, with higher

For peer review only

variability in Lao PDR and PNG (intraclass coefficient [ICC] 0.1) and lower variability in Mongolia (ICC 0.01).

Missing data

We will describe the number of participants with missing data on individual variables and compare the characteristics of those with and without missing data to determine whether there is evidence of systematic differences in characteristics. If there is evidence of systematic differences between those with and without missing data, we will consider using multiple imputation to predict the distribution of the missing data in order to account for the bias due to incomplete data.[37]

CURRENT STATUS OF THE STUDY

Recruitment is ongoing and analysis of data, followed by publication of results, is expected from 2018 onwards. As of August 2017, we have recruited 1039, 481 and 3847 cases from Lao PDR, PNG and Mongolia respectively. Table 4 describes the baseline characteristics of the children recruited.

Table 4: Case characteristics by site, Lao People's Democratic Republic (PDR), Mongolia and Papua New Guinea (PNG), 2014-2017

		Lao PDR<br>n=1039<br>n (%)	Mongolia<br>n=3847<br>n (%)	PNG<br>n=481<br>n (%)
Year of recruitment	2014	365/1039 (35)	NA	NA
	2015	323/1039 (31)	885/3847 (23)	NA
	2016	281/1039 (27)	2007/3847 (52)	190/481 (40)
	2017	70/1039 (7)	955/3847 (25)	291/481 (60)
Age group	< 12 months	432/1038 (42)	1481/3847 (39)	258/481 (54)
	12-23 months	346/1038 (33)	1250/3847 (32)	123/481 (25)
	>=24 months	260/1038 (25)	1116/3847 (29)	100/481 (21)
Gender	Male	591/1039 (57)	2079/3847 (54)	278/481 (58)

ETHICS AND DISSEMINATION

Prospective participants will be fully informed about the potential risks and benefits of participation and written informed consent will be obtained prior to recruitment. The study is being conducted according to protocols approved by the following ethics committees: Lao PDR Ministry of Health National Ethics Committee for Health Research (057/2013 NECHR), Oxford Tropical Research Ethics Committee (1050-13), Mongolian National Ethics Committee for Health Research, the WHO Regional Office for the Western Pacific (WPRO) Ethics Review Committee (2013.30.LAO.2.EPI, Mongolia), PNG IMR Institutional Review Board (1510), Government of PNG Medical Research Advisory

For peer review only

Committee (15.18), and the Royal Children's Hospital/MCRI Human Research Ethics Committee (33177B, 33203E).

We plan on disseminating results to relevant stakeholders within Lao PDR, PNG and Mongolia, as well as submitting our findings for publication in relevant peer-reviewed journals and conferences.

DISCUSSION

The ability for low- and middle-income countries to monitor the indirect effects of PCV is critical. Maximising indirect effects is important because these effects comprise a substantial proportion of overall PCV impact and increase the cost-effectiveness of the vaccine.[38] Indirect protection is particularly important for individuals who are unable to be vaccinated or who have poor vaccine responses, such as infants too young to be vaccinated and the elderly. In addition, the Bill & Melinda Gates Foundation are supporting studies looking into the effectiveness of reduced dose schedules (1+1). This reduced dose schedule needs to maintain indirect effects following PCV introduction and this surveillance method provides a mechanism to determine when herd immunity has been achieved and whether it is maintained.[39] Furthermore, the ability to determine whether substantial herd immunity has been achieved may help to identify settings which are appropriate for introduction of reduced dose or modified schedules.

The results of our study will address the scarcity of literature determining the PCV coverage required to achieve substantial indirect effects, especially in low- and middle-income countries. The inclusion of three sites, which have contrasting vaccine schedules, baseline intensities of pneumococcal carriage and health-care systems, will also enable us to determine whether this vaccine coverage threshold differs by site and explore factors which may modify the vaccine coverage required to achieve indirect effects.

In this proposal, we describe a novel surveillance method to measure indirect effects. Although the isolation of a particular pneumococcal serotype from the nasopharynx of a child with ARI is not necessarily indicative of the serotype causing pneumococcal disease, reduced detection of VT pneumococci is likely to reflect reductions in disease due to VT pneumococci. Furthermore, our proposed analysis methods enable estimation of indirect effects in the absence of baseline pre-PCV data. This will be relevant for many low- and middle-income countries that have little or no baseline data and are considering options for surveillance to accompany the introduction of PCV.

Another key strength of our methods is the use of consistent molecular serotyping microarray methods across all three sites, enabling sensitive detection of multiple serotype carriage and ascertainment of serotype-specific density.[28] Conventional pneumococcal carriage studies typically detect the presence of a single serotype and may overlook the presence of vaccine serotypes occurring a lower densities. The results of this study will also add to the limited literature on the effects of PCV on vaccine-type carriage density, which may affect likelihood of transmission. [40]

Our proposed methods have several potential limitations. Firstly, there are significant challenges in determining accurate estimates of PCV coverage in resource-limited settings. In Mongolia and Lao

For peer review only

PDR, where we are using administrative data, our estimates rely on the availability of accurate data about vaccine doses administered (numerator) as well as population estimates for the target age group (denominator). In both settings, we are conducting audits to assess the reliability of numerator data. Regarding denominator data, we are fortunate that a recent population census was conducted in Lao PDR in March 2015. However, we will be auditing denominator data in Mongolia, where population estimates are affected by large seasonal population movements between rural and urban settings. In PNG, the validity of the community surveys depends on representative sampling. However survey participation can vary and is affected by season (related to farming practices), as well as community trust and understanding of the study. To maximise participation, we conducted mobile health clinics alongside the community surveys.

The second main limitation relates to detecting pneumococcal carriage in our study population of children hospitalised with respiratory infection. In this population, carriage detection may be affected by the prior use of antibiotics.[41 42] To address this, we have, where possible, aimed to recruit patients at admission, prior to receiving antibiotics or documented prior antibiotic use, which will be taken into account during analysis.

Thirdly, there are variations in methods and setting across the three sites. This limitation may impact on the comparability of our results across the three sites. The reason for the differences in methods between sites is that our study is built on pre-existing studies (pneumonia aetiology studies in Lao PDR and PNG, and a vaccine impact study in Mongolia) with established protocols.

To conclude, the results of this study will provide important feedback to national policy makers about the effects of newly introduced PCV programs in Lao PDR, Mongolia, and PNG. The results will help us understand the determinants of indirect effects and therefore guide strategies to maximise them. In particular, the results will also inform global policy about the vaccine coverage required to achieve substantial indirect effects in settings with different epidemiological characteristics of pneumococcal disease and carriage and will maximise the cost-effectiveness of the vaccine programs.

Acknowledgements:

We would like to acknowledge the Ministries of Health of Lao PDR and Mongolia, LOMWRU, PNG IMR and WHO and support from the Victorian Government's Operational Infrastructure Support Program. We would also like to thank study staff, laboratory staff and participating families.

The PneuCAPTIVE protocol development group includes the authors of the paper listed in the by-line and the following: Dashtseren Luvsantseren (NCCD, Ulaanbaatar, Mongolia), Bujinlkham (NCCD, Ulaanbaatar, Mongolia), Mukhchuluun Ulziibayar (NCCD, Ulaanbaatar, Mongolia), Dashpagam (NCCD, Ulaanbaatar, Mongolia), Audrey Dubot-Pérès (LOMHWRU, Vientiane, Lao PDR), Keodomphone Vilavong (LOMHWRU, Vientiane, Lao PDR), Anisone Chanthongthip (LOMHWRU, Vientiane, Lao PDR), Syladeth Chanthaphone (LOMHWRU, Vientiane, Lao PDR), Joycelyn Sapura (PNG IMR, Goroka, PNG), John Kave (PNG IMR, Goroka, PNG), Tonny Kumani (PNG IMR, Goroka, PNG), Wendy Kirarock (PNG IMR, Goroka, PNG).

For peer review only

Author's contributions: FR conceived the idea and designed the study. JL, SD, KF, PN, RL, RP, AX, DD and FR supported the development of country-specific protocols and study implementation in Lao PDR. CCB, RF, DL, WP and FR supported the development of country-specific protocols and study implementation in PNG. TM, SLV, CVM, KM, JC and FR supported the development of country-specific protocols and study implementation in Mongolia. CS, ED and JH devised the microbiological approach and laboratory protocols. JC, CN, RA, and FR devised the analysis plan. JC and FR drafted the manuscript. All authors provided feedback to the draft manuscript and have read and approved the final version.

Funding statement: This work is supported by the Bill & Melinda Gates Foundation grant number (OPP1115490). JC is completing a PhD at The University of Melbourne, funded by an Australian Government Research Training Program scholarship.

Competing interests: None declared.

REFERENCES

1. Wang H, Bhutta ZA, Coates MM, et al. Global, regional, national, and selected subnational levels of stillbirths, neonatal, infant, and under-5 mortality 1980-2015: a systematic analysis for the Global Burden of Disease Study 2015. *The Lancet* 2016;388(10053):1725-74. doi: 10.1016/S0140-6736(16)31575-6

2. Hill PC, Townend J, Antonio M, et al. Transmission of Streptococcus pneumoniae in rural Gambian villages: a longitudinal study. *Clinical Infectious Diseases* 2010;50(11):1468-76. doi: http://dx.doi.org/10.1086/652443

3. Hussain M, Melegaro A, Pebody RG, et al. A longitudinal household study of Streptococcus pneumoniae nasopharyngeal carriage in a UK setting. *Epidemiology and Infection* 2005;133(5):891-98. doi: http://dx.doi.org/10.1017/S0950268805004012

4. Gray BM, Converse GM, 3rd, Dillon HC, Jr. Epidemiologic studies of Streptococcus pneumoniae in infants: acquisition, carriage, and infection during the first 24 months of life. *The Journal of infectious diseases* 1980;142(6):923-33. [published Online First: 1980/12/01]

5. Varon E, Cohen R, Bechet S, et al. Invasive disease potential of pneumococci before and after the 13-valent pneumococcal conjugate vaccine implementation in children. *Vaccine* 2015;33(46):6178-85.

6. Johnson HL, Deloria-Knoll M, Levine OS, et al. Systematic evaluation of serotypes causing invasive pneumococcal disease among children under five: the pneumococcal global serotype project. *PLoS Med* 2010;7(10) doi: 10.1371/journal.pmed.1000348 [published Online First: 2010/10/20]

7. Loo JD, Conklin L, Fleming-Dutra KE, et al. Systematic review of the effect of pneumococcal conjugate vaccine dosing schedules on prevention of pneumonia. *The Pediatric infectious disease*

For peer review only

journal 2014;33 Suppl 2:S140-51. doi: 10.1097/inf.0000000000000082 [published Online First: 2013/12/18]

8. Conklin L, Loo JD, Kirk J, et al. Systematic review of the effect of pneumococcal conjugate vaccine dosing schedules on vaccine-type invasive pneumococcal disease among young children. *The Pediatric infectious disease journal* 2014;33 Suppl 2:S109-18. doi: 10.1097/inf.0000000000000078 [published Online First: 2013/12/18]

9. Le Saux N. Pneumococcal conjugate vaccines for preventing otitis media. *Paediatrics & Child Health* 2016;21(2):89-90.

10. Loo JD, Conklin L, Fleming-Dutra KE, et al. Systematic review of the indirect effect of pneumococcal conjugate vaccine dosing schedules on pneumococcal disease and colonization. *Pediatric Infectious Disease Journal* 2014;33 Suppl 2:S161-71.

11. Direct and indirect effects of routine vaccination of children with 7-valent pneumococcal conjugate vaccine on incidence of invasive pneumococcal disease--United States, 1998-2003. *MMWR Morbidity and mortality weekly report* 2005;54(36):893-7. [published Online First: 2005/09/16]

12. Tsaban G, Ben-Shimol S. Indirect (herd) protection, following pneumococcal conjugated vaccines introduction: A systematic review of the literature. *Vaccine* 2017;35(22):2882-91. doi: 10.1016/j.vaccine.2017.04.032 [published Online First: 2017/04/30]

13. Loughlin AM, Hsu K, Silverio AL, et al. Direct and indirect effects of PCV13 on nasopharyngeal carriage of PCV13 unique pneumococcal serotypes in Massachusetts' children. *The Pediatric infectious disease journal* 2014;33(5):504-10. doi: 10.1097/inf.0000000000000279 [published Online First: 2014/03/29]

14. Grant LR, Hammitt LL, O'Brien SE, et al. Impact of the 13-valent pneumococcal conjugate vaccine on pneumococcal carriage among American Indians. *The Pediatric infectious disease journal* 2016;35(8):907-14. doi: 10.1097/inf.0000000000001207 [published Online First: 2016/05/14]

15. International Vaccine Access Center - John Hopkins Bloomberg School of Public Health. VIEW-hub 2016 [accessed 17/12/2016.

16. Shiri T, Datta S, Madan J, et al. Indirect effects of childhood pneumococcal conjugate vaccination on invasive pneumococcal disease: a systematic review and meta-analysis. *The Lancet Global Health* 2017;5(1):e51-e59. doi: 10.1016/s2214-109x(16)30306-0

17. Mackenzie GA, Hill PC, Jeffries DJ, et al. Effect of the introduction of pneumococcal conjugate vaccination on invasive pneumococcal disease in The Gambia: a population-based surveillance study. *The Lancet Infectious diseases* 2016;16(6):703-11. doi: 10.1016/s1473-3099(16)00054-2 [published Online First: 2016/02/22]

For peer review only

18. von Gottberg A, de Gouveia L, Tempia S, et al. Effects of vaccination on invasive pneumococcal disease in South Africa. *The New England journal of medicine* 2014;371(20):1889-99. doi: 10.1056/NEJMoa1401914 [published Online First: 2014/11/12]

19. Rodgers GL, Klugman KP. Surveillance of the impact of pneumococcal conjugate vaccines in developing countries. *Human Vaccines and Immunotherapeutics* 2016;12(2):417-20. doi: http://dx.doi.org/10.1080/21645515.2015.1057671

20. Gladstone RA, Jefferies JM, Tocheva AS, et al. Five winters of pneumococcal serotype replacement in UK carriage following PCV introduction. *Vaccine* 2015;33(17):2015-21.

21. Dunne EM, Manning J, Russell FM, et al. Effect of pneumococcal vaccination on nasopharyngeal carriage of Streptococcus pneumoniae, Haemophilus influenzae, Moraxella catarrhalis, and Staphylococcus aureus in Fijian children. *J Clin Microbiol* 2012;50(3):1034-8. doi: 10.1128/jcm.06589-11 [published Online First: 2011/12/16]

22. Mosser JF, Grant LR, Millar EV, et al. Nasopharyngeal carriage and transmission of Streptococcus pneumoniae in American Indian households after a decade of pneumococcal conjugate vaccine use.[Erratum appears in PLoS One. 2014;9(3):e93878]. *PLoS ONE [Electronic Resource]* 2014;9(1):e79578.

23. Lehmann D, Gratten M, Montgomery J. Susceptibility of pneumococcal carriage isolates to penicillin provides a conservative estimate of susceptibility of invasive pneumococci. *The Pediatric infectious disease journal* 1997;16(3):297-305. [published Online First: 1997/03/01]

24. Nguyen VH, Dubot-Peres A, Russell FM, et al. Acute respiratory infections in hospitalized children in Vientiane, Lao PDR - the importance of Respiratory Syncytial Virus. *Scientific reports* 2017;7(1):9318. doi: 10.1038/s41598-017-09006-6 [published Online First: 2017/08/26]

25. Blyth CC, Ford R, Sapura J, et al. Childhood pneumonia and meningitis in the Eastern Highlands Province, Papua New Guinea in the era of conjugate vaccines: study methods and challenges. *Pneumonia (Nathan Qld)* 2017;9:5. doi: 10.1186/s41479-017-0029-y [published Online First: 2017/07/14]

26. Satzke C, Turner P, Virolainen-Julkunen A, et al. Standard method for detecting upper respiratory carriage of Streptococcus pneumoniae: updated recommendations from the World Health Organization Pneumococcal Carriage Working Group. *Vaccine* 2013;32(1):165-79. doi: 10.1016/j.vaccine.2013.08.062 [published Online First: 2013/12/18]

27. Dunne EM, Manning J, Russell FM, et al. Effect of Pneumococcal Vaccination on Nasopharyngeal Carriage of Streptococcus pneumoniae, Haemophilus influenzae, Moraxella catarrhalis, and Staphylococcus aureus in Fijian Children. *Journal of Clinical Microbiology* 2012;50(3):1034-38. doi: 10.1128/JCM.06589-11

For peer review only

28. Satzke C, Dunne EM, Porter BD, et al. The PneuCarriage Project: A Multi-Centre Comparative Study to Identify the Best Serotyping Methods for Examining Pneumococcal Carriage in Vaccine Evaluation Studies. *PLoS Medicine / Public Library of Science* 2015;12(11):e1001903; discussion e03.

29. Newton R, Hinds J, Wernisch L. Empirical Bayesian models for analysing molecular serotyping microarrays. *BMC bioinformatics* 2011;12:88. doi: 10.1186/1471-2105-12-88 [published Online First: 2011/04/02]

30. Scott P, Rutjes AW, Bermetz L, et al. Comparing pneumococcal conjugate vaccine schedules based on 3 and 2 primary doses: systematic review and meta-analysis. *Vaccine* 2011;29(52):9711-21. doi: 10.1016/j.vaccine.2011.07.042 [published Online First: 2011/08/09]

31. Chan J. High agreement between the new Mongolian electronic immunisation register and clinic immunisation records: a health centre based audit. *Western Pacific Surveillance and Response Journal* 2017;In press

32. Deen J, Ali M, Sack D. Methods to assess the impact of mass oral cholera vaccination campaigns under real field conditions. *PLoS ONE* 2014;9(2):e88139. doi: 10.1371/journal.pone.0088139

33. Khatib AM, Ali M, von Seidlein L, et al. Effectiveness of an oral cholera vaccine in Zanzibar: findings from a mass vaccination campaign and observational cohort study. *The Lancet Infectious diseases* 2012;12(11):837-44. doi: 10.1016/s1473-3099(12)70196-2 [published Online First: 2012/09/08]

34. Moore MR, Hyde TB, Hennessy TW, et al. Impact of a conjugate vaccine on community-wide carriage of nonsusceptible Streptococcus pneumoniae in Alaska. *Journal of Infectious Diseases* 2004;190(11):2031-8.

35. Usuf E, Bottomley C, Adegbola RA, et al. Pneumococcal carriage in sub-Saharan Africa - A systematic review. *PLoS ONE* 2014;9 (1) (no pagination)(e85001) doi: http://dx.doi.org/10.1371/journal.pone.0085001

36. Aho C, Michael A, Yoannes M, et al. Limited impact of neonatal or early infant schedules of 7-valent pneumococcal conjugate vaccination on nasopharyngeal carriage of Streptococcus pneumoniae in Papua New Guinean children: A randomized controlled trial. *Vaccine Reports* 2016;6:36-43. doi: http://dx.doi.org/10.1016/j.vacrep.2016.08.002

37. Sterne JAC, White IR, Carlin JB, et al. Multiple imputation for missing data in epidemiological and clinical research: potential and pitfalls. *Bmj* 2009;338 doi: 10.1136/bmj.b2393

For peer review only

38. Isaacman DJ, Strutton DR, Kalpas EA, et al. The impact of indirect (herd) protection on the cost-effectiveness of pneumococcal conjugate vaccine. *Clinical therapeutics* 2008;30(2):341-57. doi: 10.1016/j.clinthera.2008.02.003 [published Online First: 2008/03/18]

39. Rodgers GL, Klugman KP. A new paradigm in pneumococcal conjugate vaccination: moving from individual to herd protection. *International journal of infectious diseases : IJID : official publication of the International Society for Infectious Diseases* 2017 doi: 10.1016/j.ijid.2017.04.015

40. Roca A, Bottomley C, Hill PC, et al. Effect of age and vaccination with a pneumococcal conjugate vaccine on the density of pneumococcal nasopharyngeal carriage. *Clinical Infectious Diseases* 2012;55(6):816-24.

41. Abdullahi O, Karani A, Tigoi CC, et al. The prevalence and risk factors for pneumococcal colonization of the nasopharynx among children in Kilifi District, Kenya. *PLoS One* 2012;7(2):e30787. doi: 10.1371/journal.pone.0030787 [published Online First: 2012/03/01]

42. Alpkvist H, Athlin S, Naucler P, et al. Clinical and Microbiological Factors Associated with High Nasopharyngeal Pneumococcal Density in Patients with Pneumococcal Pneumonia. *PLoS One* 2015;10(10):e0140112. doi: 10.1371/journal.pone.0140112 [published Online First: 2015/10/16]

For peer review only

BMJ Open

DETERMINING THE PNEUMOCOCCAL CONJUGATE VACCINE COVERAGE REQUIRED FOR INDIRECT PROTECTION AGAINST VACCINE-TYPE PNEUMOCOCCAL CARRIAGE IN LOW- AND MIDDLE-INCOME COUNTRIES: A PROTOCOL FOR A PROSPECTIVE OBSERVATIONAL STUDY

Journal:	*BMJ Open*
Manuscript ID	bmjopen-2018-021512.R1
Article Type:	Protocol
Date Submitted by the Author:	20-Feb-2018
Complete List of Authors:	Chan, Jocelyn; Murdoch Childrens Research Institute, Pneumococcal Research Group; The University of Melbourne, Department of Paediatrics<br>Nguyen, Cattram; Murdoch Childrens Research Institute, Pneumococcal Research; The University of Melbourne, Department of Paediatrics<br>Lai, Jana; Murdoch Childrens Research Institute, Pneumococcal Research Group<br>Dunne, Eileen; Murdoch Childrens Research Institute, Pneumococcal Research Group; The University of Melbourne, Department of Paediatrics<br>Andrews, Ross; Menzies School of Health Research, Charles Darwin University, Global & Tropical Health Division; Australian National University, National Centre for Epidemiology & Population Health<br>Blyth, Christopher C.; University of Western Australia, School of Medicine; Princess Margaret Hospital, Department of Infectious Diseases<br>Datta, Siddhartha; World Health Organization<br>Fox, Kim; World Health Organization, Regional Office for the Western Pacific<br>Ford, Rebecca ; Papua New Guinea Institute of Medical Research<br>Hinds, Jason; St George's- University of London, Institute for Infection and Immunity; London Bioscience Innovation Centre, BUGS Bioscience<br>La Vincente, Sophie; Murdoch Childrens Research Institute, Pneumococcal Research Group<br>Lehmann, Deborah; Telethon Kids Institute, University of Western Australia, Wesfarmers Centre for Vaccines and Infectious Diseases<br>Lim, Ruth; Murdoch Childrens Research Institute, Pneumococcal Research Group<br>Mungun, Tuya ; Ministry of Health, National Center of Communicable Diseases (NCCD)<br>Newton, Paul ; Lao-Oxford-Mahosot Hospital-Wellcome Trust Research Unit , Microbiology Laboratory, Mahosot Hospital; University of Oxford, Centre for Tropical Medicine and Global Health<br>Phetsouvanh, Rattanaphone; Lao-Oxford-Mahosot Hospital-Wellcome Trust Research Unit, Microbiology Laboratory, Mahosot Hospital; University of Oxford, Centre for Tropical Medicine and Global Health<br>Pomat, Willie; PNG Institute of Medical Research, Infection and Immunity; Telethon Kids Institute, University of Western Australia, Wesfarmers Centre for Vaccines and Infectious Diseases<br>Xeuatvongsa, Anonh; Ministry of Health, National Immunization

	Programme<br>von Mollendorf, Claire; Murdoch Childrens Research Institute, Pneumococcal Research Group; The University of Melbourne, Department of Paediatrics<br>Dance, David; Lao-Oxford-Mahosot Hospital-Wellcome Trust Research Unit, Microbiology Laboratory, Mahosot Hospital; University of Oxford, Centre for Tropical Medicine and Global Health<br>Satzke, Catherine; Murdoch Childrens Research Institute, Pneumococcal Research; The University of Melbourne at the Peter Doherty Institute for Infection and Immunity, Department of Microbiology and Immunology<br>Muholland , Kim; Murdoch Childrens Research Institute, Pneumococcal Research Group; London School of Hygiene and Tropical Medicine, Department of Infectious Disease Epidemiology<br>Russell, F. M.; Murdoch Childrens Research Institute, Pneumococcal Research Group; The University of Melbourne, Centre for International Child Health, Department of Paediatrics
**Primary Subject Heading**:	Global health
Secondary Subject Heading:	Infectious diseases, Epidemiology
Keywords:	Public health < INFECTIOUS DISEASES, Paediatric infectious disease & immunisation < PAEDIATRICS, Respiratory infections < THORACIC MEDICINE

SCHOLARONE™
Manuscripts

For peer review only

DETERMINING THE PNEUMOCOCCAL CONJUGATE VACCINE COVERAGE REQUIRED FOR INDIRECT PROTECTION AGAINST VACCINE-TYPE PNEUMOCOCCAL CARRIAGE IN LOW- AND MIDDLE-INCOME COUNTRIES: A PROTOCOL FOR A PROSPECTIVE OBSERVATIONAL STUDY

Authors:

Chan J[1,2], Nguyen CD[1,2], Lai JYR[1], Dunne EM[1,2], Andrews R[3,4], Blyth CC[5,6], Datta S[7], Fox K[8], Ford R[9], Hinds J[10,11], La Vincente S[1] , Lehmann D[12], Lim R[1], Mungun T[13], Newton PN[14,15], Phetsouvanh R[14,15*], Pomat W[9,12], Xeuatvongsa A[16], von Mollendorf C[1,2], Dance DAB[14,15] Satzke C[1,17], Mulholland K[1,18], Russell FM[1,19] for the PneuCAPTIVE Protocol Group

1. Pneumococcal Research Group, Murdoch Children's Research Institute, Melbourne, Australia.
2. Department of Paediatrics, The University of Melbourne, Melbourne, Australia.
3. Global & Tropical Health Division, Menzies School of Health Research, Charles Darwin University, Darwin, Australia.
4. National Centre for Epidemiology & Population Health, Australian National University, Canberra, Australia
5. University of Western Australia, School of Medicine, Perth, Australia;
6. Department of Infectious Diseases, Princess Margaret Hospital, Perth, Australia;
7. World Health Organization, Vientiane, Lao People's Democratic Republic
8. Regional Office for the Western Pacific, World Health Organization, Manila, Philippines.
9. Papua New Guinea Institute of Medical Research, Infection and Immunity Unit, Goroka, Papua New Guinea.
10. Institute for Infection and Immunity, St George's- University of London, London, United Kingdom.
11. BUGS Bioscience, London Bioscience Innovation Centre, London, United Kingdom.
12. Wesfarmers Centre for Vaccines and Infectious Diseases, Telethon Kids Institute, University of Western Australia, Perth, Australia.
13. National Center of Communicable Diseases (NCCD), Ministry of Health, Ulaanbaatar, Mongolia.
14. Lao-Oxford-Mahosot Hospital-Wellcome Trust Research Unit (LOMHWRU), Microbiology Laboratory, Mahosot Hospital, Vientiane, Lao PDR.
15. Centre for Tropical Medicine and Global Health, University of Oxford, Oxford, United Kingdom.
16. National Immunization Programme, Ministry of Health, Vientiane, Lao PDR.
17. Department of Microbiology and Immunology, The University of Melbourne at the Peter Doherty Institute for Infection and Immunity, Melbourne, Australia
18. Department of Infectious Disease Epidemiology, London School of Hygiene and Tropical Medicine, London, United Kingdom.

For peer review only

For peer review only - http://bmjopen.bmj.com/site/about/guidelines.xhtml

19. Centre for International Child Health, Department of Paediatrics, The University of Melbourne, Melbourne, Australia.

*Deceased

Corresponding author: Jocelyn Chan

Pneumococcal Research Group,
Murdoch Children's Research Institute
50 Flemington Road, Parkville 3052 VIC
Australia

Email: jocelyn.chan@mcri.edu.au

Telephone: +61 3 9345 4968

For peer review only

ABSTRACT

Introduction

Pneumococcal conjugate vaccines (PCVs) prevent disease through both direct protection of vaccinated individuals, and indirect protection of unvaccinated individuals by reducing nasopharyngeal (NP) carriage and transmission of vaccine-type (VT) pneumococci. While the indirect effects of PCV vaccination are well described, the PCV coverage required to achieve the indirect effects is unknown. We will investigate the relationship between PCV coverage and vaccine-type carriage among under-vaccinated children using hospital-based NP pneumococcal carriage surveillance at three sites in the Asia-Pacific region.

Methods and analysis

We are recruiting cases, defined as children aged 2-59 months admitted to participating hospitals with acute respiratory infection in Lao People's Democratic Republic, Mongolia and Papua New Guinea. Thirteen-valent PCV (PCV13) status is obtained from written records. NP swabs are collected according to standard methods, screened using *lytA* qPCR and serotyped by microarray. Village-level vaccination coverage, for the resident communities of the recruited cases, is determined using administrative data or community survey. Our analysis will investigate the relationship between VT carriage among under-vaccinated cases (indirect effects) and vaccine coverage using generalised estimating equations.

Ethics and dissemination

Ethical approval has been obtained from the relevant ethics committees at participating sites. The results are intended for publication in open-access peer-reviewed journals and will demonstrate methods suitable for low- and middle-income countries to monitor vaccine impact, and inform vaccine policy makers about the PCV coverage required to achieve indirect protection.

STRENGTHS AND LIMITATIONS

- This protocol describes a method for monitoring the indirect effects of pneumococcal conjugate vaccines (PVCs) on pneumococcal carriage in low- and middle-income countries, in the absence of baseline pre-PCV data,
- This method does not measure indirect protection against disease. However, a reduction in VT carriage among undervaccinated cases indicates likely reductions in VT disease, since carriage is a precursor for disease The use of molecular serotyping microarray enables detection of multiple serotype carriage and serotype-specific carriage density
- The inclusion of three sites, which have contrasting vaccine schedules and pneumococcal epidemiology, will enable us to explore factors which may modify the vaccine coverage required to achieve indirect effects however variations in methods and settings may impact on the comparability of our results across the three sites

For peer review only - http://bmjopen.bmj.com/site/about/guidelines.xhtml

INTRODUCTION

Infections due to *Streptococcus pneumoniae* (the pneumococcus), including pneumonia, meningitis and septicaemia, are a leading cause of morbidity and mortality among children and elderly, especially in low- and middle-income countries.[1] The main reservoir for the pneumococcus is the human nasopharynx. Pneumococcal carriage peaks in young children, an important group for transmission of pneumococci to older age groups,[2 3] and is a precursor for disease.[4] There are over 90 known serotypes of pneumococci, with differing capacities for causing disease.[5]

The introduction of the pneumococcal conjugate vaccine (PCV) has resulted in substantial reductions in pneumococcal disease in many settings.[6] These reductions are mediated by direct effects on vaccinated children as well as indirect effects on under-vaccinated children and adults through a reduction in transmission and subsequent nasopharyngeal (NP) carriage of pneumococcal serotypes included in the vaccine.[7-10] The indirect effects account for a substantial component of the overall vaccine effect. Following the introduction of the 7-valent PCV (PCV7) into the routine vaccination program in the USA, twice as many invasive cases were prevented through indirect effects compared to direct effects.[11]

The magnitude of indirect effects following introduction of PCV varies considerably by setting. Reductions in invasive pneumococcal disease (IPD) among adults ranged from 8.8% among adults in Denmark three years after PCV implementation, to 70% reduction among adults in Taiwan, seven years after PCV implementation.[12] A review of the literature found that higher rates of coverage, higher baseline rates of pneumococcal disease and greater time elapsed since PCV introduction were all associated with greater degrees of indirect effects.[12] However, the majority of these studies were conducted in high-income country settings using a similar vaccine schedule, with relatively high levels of vaccine coverage.

The threshold of vaccination coverage required to achieve significant indirect PCV effects on either pneumococcal carriage or disease outcomes are not well understood. Studies of NP carriage indicate that there is variability in the coverage required to achieve significant indirect effects. Two studies in the US examined this question for the 13-valent PCV (PCV13) that superseded 7-valent (PCV7) immunisation. A vaccine coverage of 58% among American Indian children in south-western USA and 75% coverage among children in Massachusetts resulted in a 50% decline in the prevalence of carriage of six PCV13 serotypes (i.e. PCV13 types not included in PCV7) carried by under-vaccinated children.[13 14] The US uses a 3+1 schedule (three primary doses with a booster) and had a catch-up program up to five years of age. Many low- and middle-income countries utilise a 3+0 schedule. [15] More data are needed from a range of low- and middle-income countries to determine the vaccination coverage required to maximise the indirect effects of PCVs, and to determine factors which may modify the vaccine coverage required for indirect effects, such as baseline carriage prevalence, indicating intensity of transmission, vaccine schedules and use of catch-up campaigns.

Existing systematic reviews indicate that vaccine schedules do not impact on the degree of indirect effects on IPD, however the studies included in these reviews predominantly use a 3+1 schedule or a

For peer review only - http://bmjopen.bmj.com/site/about/guidelines.xhtml

2+1 schedule (two primary doses with a booster) and have limited studies using PCV13.[12 16] Few studies using the 3+0 schedule (three primary doses with no booster) are available in the published literature, despite the 3+0 schedule being widely used, especially in low- and middle-income countries. A recent study from Australia, the only high-income country to use the 3+0 schedule, concluded that the booster dose may be important for obtaining indirect protection because of the lower indirect effects observed compared to other high-income country settings.[17]

Despite the gradual introduction of PCVs in low- and middle-income countries over the last decade, there have been few studies published on the impacts of PCV in these settings.[18 19] This is because the predominant method for assessing vaccine impact, IPD surveillance, is costly and resource-intensive to establish – requiring laboratory capacity as well as the collection of large numbers of samples, obtained using aseptic techniques, in order to detect a relatively rare outcome.[20] Furthermore, baseline data prior to vaccine introduction required for impact evaluations are often not available in these settings.

In the absence of IPD surveillance, low- and middle-income countries require a method to evaluate direct and indirect vaccine effects. We propose using NP carriage surveillance in children hospitalised with acute respiratory infection to monitor the indirect effects of PCV13 in the study called PneuCAPTIVE (PNEUmococcal CArriage in Pneumonia To Investigate Vaccine Effects).

In this context, we refer to "carriage" as the detection of pneumococci in the nasopharynx of a child with acute respiratory infection. A reduction in vaccine-type (VT) pneumococcal carriage will likely reflect reductions in disease due to VT pneumococci, since carriage is a precursor to disease.[4] Existing studies among healthy children have demonstrated reductions in VT carriage following PCV introduction, while overall carriage remains stable due to replacement with non-vaccine type (NVT) carriage.[21 22]

We aim to determine the PCV13 coverage required to demonstrate substantial indirect effects of PCV using NP carriage surveillance in children hospitalised with acute respiratory infection (ARI) in three settings within the Asia-Pacific region. We are focussing on children under five years of age because studies have shown that children are the main reservoir for pneumococci, and reductions in transmission within this age group are likely to result in reductions in VT carriage among older age groups. [2 3 23]

As this is a novel method for determining indirect effects, we will also aim to determine whether changes in patterns of VT and NVT pneumococcal carriage among children with ARI are reflective of changes in serotypes circulating in the community, noting that carriage in our cohort may be more reflective of serotypes causing disease. [5 24]

OBJECTIVES

Our objectives are to: (1) investigate the relationship between PCV13 coverage and VT carriage among under-vaccinated cases, defined as children (indirect effects) aged 2-59 months with an ARI in Lao People's Democratic Republic (Lao PDR), Mongolia and Papua New Guinea (PNG); (2) describe

For peer review only - http://bmjopen.bmj.com/site/about/guidelines.xhtml

monthly trends in VT carriage prevalence among cases and contacts. Contacts are defined as children 0-59 months of age, who have slept in the same house as or played with the case during the preceding three weeks; (3) investigate the relationship between PCV13 coverage and VT carriage among under-vaccinated contacts (indirect effects) and caregivers living in the community; and (4) compare the PCV13 coverage required to demonstrate indirect effects of PCV13 by site and determine the degree to which site-specific factors, such as baseline pneumococcal carriage rates and densities, vaccine schedule and use of catch-up campaigns, account for differences in the PCV13 coverage required to demonstrate indirect effects.

METHODS

Study design

We are conducting prospective hospital-based observational studies in Lao PDR, Mongolia and PNG. We are recruiting children 2-59 months of age presenting with ARI and obtaining NP swabs to determine prevalence and density of VT carriage. We are determining the PCV13 status of each case using written record. Recruitment will occur over at least three years and up to five years post-PCV13 introduction at each site.

Concurrently, we are determining vaccination coverage at the resident village or subdistrict of each recruited case, using either administrative data or vaccination coverage surveys.

Study settings

Lao PDR site

The Lao PDR PneuCAPTIVE study is embedded within a hospital-based study of ARI aetiology, in collaboration with Lao Oxford Mahosot Hospital-Wellcome Trust Research Unit (LOMWRU), the World Health Organization (WHO) and the Lao PDR Ministry of Health [25]. PCV13 was introduced in October 2013, using a 3+0 schedule at 6, 10 and 14 weeks of age and a catch-up program up to 12 months of age (Table 1). We will recruit cases at Mahosot Hospital, one of the largest paediatric referral hospitals in Vientiane, the capital of Lao PDR.

Mongolia site

The Mongolian PneuCAPTIVE study is embedded within a hospital-based paediatric pneumonia surveillance to determine vaccine impact which is conducted in partnership between Murdoch Children's Research Institute (MCRI), WHO and the Mongolian Ministry of Health. PCV13 was introduced in June 2016, using a modified 2+1 schedule at 2, 4 and 9 months of age, within the two 'Phase 1' districts within Ulaanbaatar, the capital of Mongolia as part of a phased introduction (Table 1), with a catch-up program of two doses one month apart for those up to 24 months of age. We are recruiting cases, residing in the two phase 1 districts, at the two district hospitals and the tertiary referral paediatric hospital for Mongolia, the Maternal and Child Hospital (MCH).

PNG site

The PNG PneuCAPTIVE study represents a collaboration between the PNG Institute of Medical Research (IMR), Telethon Kids Institute, the University of Western Australia and MCRI. It is an extension of a pneumonia aetiology study that commenced in 2013 [26]. PCV13 was introduced to PNG

For peer review only - http://bmjopen.bmj.com/site/about/guidelines.xhtml

in October 2014 using a 3+0 schedule at 1, 2 and 3 months of age (Table 1), however was not widely distributed in the Eastern Highlands Province until late 2015. We are recruiting cases at the Eastern Highlands Provincial Hospital, the major referral hospital for the Eastern Highland Province, as well as nearby clinics in Goroka, the capital of the Eastern Highlands Province. In PNG, we are also recruiting caregivers, as well as contacts, defined as children 0-59 months of age, who have slept in the same house as or played with the case during the preceding three weeks. This will enable us to determine whether changes in patterns of VT pneumococcal carriage in the hospitalised cases are reflective of changes within the community, as well as to examine indirect effects in the adult age group.

Table 1: Key aspects of the 13-valent pneumococcal conjugate vaccination (PCV13) program by site, Lao People's Democratic Republic (PDR), Mongolia and Papua New Guinea (PNG)

	Lao PDR	Mongolia	PNG
Year of PCV13 introduction	Oct 2013	Jun 2016	Oct 2014
Location of PCV13 introduction	National	Two districts in Ulaanbaatar	National
PCV13 schedule	3+0 (6,10 and 14 weeks)	2+1 (2,4, and 9 months)	3+0 (1, 2 and 3 months)
Presence of a catch-up program	Catch-up of three doses up to 12 months of age	Catch-up of two doses two months apart up to 24 months of age	None

Case recruitment and data collection

Participant recruitment and data collection are consistent across the three sites; however there are some local adaptations to the protocol at each site which are summarised in Table 2. These adaptations are due to the PneuCAPTIVE study being nested within other existing studies, described above.

Cases are eligible for inclusion in the PneuCAPTIVE study if they are 2-59 months of age, and presenting with ARI (defined in Table 2 below). All cases with fever or respiratory symptoms are screened for inclusion. In Mongolia, we are restricting recruitment to patients living within the two 'Phase 1' districts which have commenced PCV13 in 2016. In PNG, we are restricting recruitment to patients living within one hour of the town as follow-up is logistically challenging.

Regarding recruitment, in Lao PDR, study staff are screening potential recruits from Monday to Friday each week. However, they are obtaining clinical information from medical records for all eligible cases, including those admitted at weekends to ensure we have a representative sample. In Mongolia, caregivers of all eligible children presenting to the hospital will be approached for recruitment as part of the larger PCV impact study. However, for the purposes of this study we will select a random sample of 33 cases per month for microbiological testing. In PNG recruitment takes place 4-5 days per week.

For peer review only - http://bmjopen.bmj.com/site/about/guidelines.xhtml

Table 2: Patient eligibility by site, Lao People's Democratic Republic (PDR), Mongolia and Papua New Guinea (PNG)

**Site**	Lao PDR	Mongolia	PNG
**Inclusion criteria**	2-59 months of age and presenting with:		
**Definition of acute respiratory infection**	Fever (parent report or measured) AND one of cough OR dyspnoea OR rhinitis OR abnormal chest auscultation	Cough OR dyspnoea AND tachypnoea* OR hypoxia OR chest indrawing	Cough AND tachypnoea* AND lower chest wall indrawing
**Exclusion criteria**	-	Lives outside Phase 1 district<br>Admitted with pneumonia in the last 14 days	Lives ≥ 1 hour outside town OR hospitalisation in past 14 days
**Recruitment site**	Inpatient setting only	Inpatient setting only	Inpatient and outpatient setting
**Sampling**	Monday-Friday	A random sample (33 per month) of all enrolled cases are selected for testing	Monday- Friday
**Sampling period**	December 2013-November 2019	November 2015 – October 2018†	April 2016 – March 2019
**Study population**	Mahosot Hospital is one of two tertiary-level paediatric hospitals in Vientiane and receives a mix of patients from urban Vientiane city, rural Vientiane province and other provinces.	The two secondary-level district hospitals and a tertiary-level Maternal and Child Health hospital service the vast majority of children in the two districts that received PCV. There are a limited number of paediatric beds at private hospitals in Ulaanbaatar.	The Eastern Highlands Provincial hospital is the sole hospital for the province. Study population includes urban and rural households within 1 hour drive of Goroka.

* Tachypnoea is defined as greater than or equal to 50 breaths per minute

† A one-year extension (until June 2019) has been sought for the Mongolian site

After determining eligibility and obtaining informed parental consent, we complete a questionnaire to obtain: demographic data, clinical data, PCV13 status, and risk factors for vaccination and NP

For peer review only - http://bmjopen.bmj.com/site/about/guidelines.xhtml

carriage, including prior antibiotic use (see analysis section below for complete list). Vaccination status is determined using written records – either parent-held immunisation records or health centre administrative records.

We then collect an NP swab according to WHO guidelines and store it in 1 ml skim milk tryptone glucose glycerol (STGG) medium. [27] Swabs are vortexed, aliquoted, and stored frozen at -80^{0}C within 8 hours of collection, and transported from all three sites to the Pneumococcal Research laboratory at MCRI on dry ice or in liquid nitrogen, where they will be stored at -80^{0}C.

Laboratory methods

All samples are screened for the presence of pneumococci using real-time quantitative PCR (qPCR) assay targeting the pneumococcal *lytA* gene.[28] Genomic DNA are extracted from 100 µl of STGG using a MagNA Pure LC Machine (Roche) using the DNA Isolation Kit III (Bacteria, Fungi) (Roche) following an enzymatic lysis treatment. The pneumococcal load is estimated by reference to a standard curve.

All swabs that are *lytA* positive or equivocal are molecular serotyped using BµG@S Senti-SP v1.5 microarray (BUGS Bioscience) as previously described. [29]

Serotype-specific pneumococcal density is calculated using the relative abundance of each serotype identified, as determined using microarray and interpreted with the assistance of a Bayesian random effects model as previously described,[29 30] and the overall pneumococcal load as determined by the *lytA* qPCR.

Key definitions

The primary outcome, VT carriage, is defined as the NP carriage of at least one pneumococcal serotype included in the PCV13 vaccine, i.e. serotypes 1, 3, 4, 5, 6A, 6B, 7F, 9V, 14, 18C, 19A, 19F, and 23F. In the context of multiple serotype carriage, VT carriage will be defined as the presence of at least one VT serotype regardless of the presence of other serotypes. A secondary outcome is VT carriage density (CFU/mL) which is defined as an aggregate of the serotype-specific density for each of the VTs carried by the case, and will be reported as a continuous variable using a log scale to account for large variations in density and a skewed distribution.

Vaccine history will be defined based on documented evidence of receiving an adequate number of PCV doses to provide a protective immune response against vaccine serotypes at least 14 days prior to study enrolment.[31] For children < 12 months of age, ’vaccinated’ is defined as two or more PCV13 doses. For children 12 months of age or older, ’vaccinated’ is defined as receipt of 2 doses in the first year of life or at least 1 dose after the age of 12 months. Conversely, a case will be defined as ‘under-vaccinated’ if they have received less than the adequate number of PCV doses (including those never vaccinated). Sensitivity analyses will be conducted using varying definitions of vaccinated including receiving at least one dose of vaccine at any age.

For peer review only - http://bmjopen.bmj.com/site/about/guidelines.xhtml

Vaccine coverage data collection

For each under-vaccinated case recruited (including those with and without VT pneumococci), we are determining their resident village or subdistrict vaccination coverage. In Mongolia and Lao PDR, we are using administrative data. The resident village or subdistrict is identified using relevant administrative codes, which are determined by local staff on enrolment. This determines the health centre's administrative boundary for the provision of immunisation services, whereas in PNG, we are conducting community surveys within 10 days of discharge in the village where the case is living (Table 3). We are surveying all children less than five years of age, from households within 5 minutes' walk of the case, since this is the group of children with whom the case is mostly likely to interact and therefore influence their carriage status.

To determine the reliability of our methods in Lao PDR, we plan on comparing our coverage estimates with a National Immunisation Survey, conducted according to WHO guidelines in 2015, when provincial level estimates from this survey become available. In Mongolia, we have validated the newly introduced electronic immunisation record against clinic health records, finding a high degree of concordance.[32] We are also in the process of validating the population registers at health centres in Mongolia.

Table 3: Vaccination coverage data by site, Lao People's Democratic Republic (PDR), Mongolia and Papua New Guinea

**Site**	Lao PDR	Mongolia	Papua New Guinea
**Source of numerator data**	Health centre records	Electronic immunisation record	Community surveys
**Source of denominator data**	Lao PDR Population and Housing Census 2015	Health centre population register*	

* All children are required to be registered at the health centre servicing their resident subdistrict in order to receive health services.

Data management

In Lao PDR and Mongolia, study staff are double-entering data using a RedCAP (Research Electronic Data Capture) and Microsoft Access (Microsoft Corporation) database, respectively. In PNG, data are checked by a monitor prior to being entered into Filemaker Pro (FileMaker Inc.). We are conducting regular double-entry discrepancy checks and logic checks using Stata Statistical Software (College Station, TX: StataCorp LLC).

Analysis

All analyses will be completed using Stata Statistical Software (College Station, TX: StataCorp LLC). We will summarise continuous variables using mean and standard deviation (or median and interquartile range for non-symmetrical data). Categorical variables will be summarised using frequency counts and percentages.

For peer review only - http://bmjopen.bmj.com/site/about/guidelines.xhtml

Objective 1: Relationship between vaccine coverage and indirect effects
We want to investigate the relationship between VT carriage and density among under-vaccinated cases (indirect effects) and subdistrict/village PCV coverage. We will use an adaptation of a method used to estimate indirect protection for an oral cholera vaccine which exploits heterogeneities in vaccine coverage at the subdistrict/village level, comparing VT carriage and density among under-vaccinated children from subdistricts/villages with differing levels of vaccine coverage.[33 34]

This will be done using multivariable models with VT carriage or density in ARI cases as the outcome variable and PCV coverage at the child's place of residence at the time of admission as the exposure variable. We will use generalised estimating equations to account for clustering at the subdistrict/village level.

To identify confounders for adjustment, we will constructed directed acyclic graphs (DAG). DAGs include all variables potentially related to exposure and outcome, connected using uni-directional arrows showing causal relationships between variables. The graph identifies potentially confounding pathways and allows investigators to determine variables that should be controlled for to obtain unbiased effect estimates. As there are likely to be unique confounders between sites, we will develop site-specific DAGs. We will use DAGitty.net (version 2.3) software to identify minimally sufficient confounding subsets for adjustment.

For each site, we will construct a similar model using overall pneumococcal carriage as the dependent variable. This model will act as a bias indicator since PCV coverage is not expected to affect levels of overall pneumococcal carriage due to replacement carriage with NVTs, although complete replacement to baseline levels can take several years.[21 35 36] Therefore, we will restrict this analysis to the latter part of the study period, when descriptive analyses indicate the replacement is complete.

To determine whether a higher PCV coverage is required to achieve indirect effects among completely un-vaccinated cases compared to under-vaccinated cases, we will conduct a sensitivity analysis among children who have never received PCV.

Objective 2: VT carriage prevalence among cases and contacts, by calendar month (PNG only)
Crude and adjusted monthly VT carriage prevalence will be estimated within rolling seven-month intervals to present smooth curves and assess trends over time. This will be done separately for cases and contacts. To account for differences in age between cases and community contacts, the carriage prevalence will be adjusted using direct standardisation (standardised to the case population over the entire study period).

Objective 3: Relationship between vaccine coverage and indirect effects among community contacts (PNG only)
To investigate whether relationship between vaccine coverage and indirect effects as observed among cases with ARI are reflective of the relationship between vaccine coverage and indirect effects in the wider community, we will apply the same model described above to under-vaccinated community contacts. We will construct multivariable models with VT carriage and density among

For peer review only - http://bmjopen.bmj.com/site/about/guidelines.xhtml

under-vaccinated community contacts as the outcome variable and PCV coverage at the child's place of residence at the time of admission as the exposure variable. We will be using the same vaccine coverage data as for the cases.

Objective 4: Comparison of vaccine coverage required for indirect effects across sites
We will compare differences in the PCV13 coverage required to demonstrate indirect effects of PCV13 qualitatively, by site and in relation to vaccine schedule and use of catch-up campaigns. Inferential statistics are unlikely to be suitable with the inclusion of only three sites and comparability between sites is limited due to variations between them.

Power calculation

Power calculations were performed using nQuery Advisor + nTerim 4.0. Calculations were based on sample size methods for logistic regression models with a continuous covariate (i.e. PCV coverage) and additional covariates, with inflation to account for clustering within villages. Power calculations assumed VT carriage prevalence of 30% in Lao PDR and 40% in Mongolia and PNG at the mean PCV coverage level,[37] and VT carriage prevalence of 20% in Lao PDR and 30% in Mongolia and PNG at one standard deviation above the mean PCV coverage level. Assuming a significance level of 0.05, allowing for adjustment using multiple covariates with an r-squared of 0.4, and that 50% of the cases are under-vaccinated, a sample size of 1200 cases per site would provide between 87% and 92% power to determine the proportion of cases carrying VT pneumococcus at varying levels of village vaccine coverage. The power calculation has been adjusted to account for clustering by village, with higher variability in Lao PDR and PNG (intraclass coefficient [ICC] 0.1) and lower variability in Mongolia (ICC 0.01).

Missing data

We will describe the number of participants with missing data on individual variables and compare the characteristics of those with and without missing data to determine whether there is evidence of systematic differences in characteristics. If there is evidence of systematic differences between those with and without missing data, we will consider using multiple imputation to predict the distribution of the missing data in order to account for the bias due to incomplete data.[38]

Patient and Public Involvement

Patients were not involved. Public health authorities in Laos and Mongolia were involved in the design and conduct of the study. In PNG, village representatives are approached to ensure that community surveys are conducted appropriately.

CURRENT STATUS OF THE STUDY

Recruitment is ongoing and analysis of data, followed by publication of results, is expected from 2018 onwards. As of August 2017, we have recruited 1039, 481 and 3847 cases from Lao PDR, PNG and Mongolia respectively. Table 4 describes the baseline characteristics of the children recruited.

For peer review only - http://bmjopen.bmj.com/site/about/guidelines.xhtml

Table 4: Case characteristics by site, Lao People's Democratic Republic (PDR), Mongolia and Papua New Guinea (PNG), 2014-2017

		Lao PDR<br>n=1039<br>n (%)	Mongolia<br>n=3847<br>n (%)	PNG<br>n=481<br>n (%)
Year of recruitment	2014	365/1039 (35)	NA	NA
	2015	323/1039 (31)	885/3847 (23)	NA
	2016	281/1039 (27)	2007/3847 (52)	190/481 (40)
	2017	70/1039 (7)	955/3847 (25)	291/481 (60)
Age group	< 12 months	432/1038 (42)	1481/3847 (39)	258/481 (54)
	12-23 months	346/1038 (33)	1250/3847 (32)	123/481 (25)
	>=24 months	260/1038 (25)	1116/3847 (29)	100/481 (21)
Gender	Male	591/1039 (57)	2079/3847 (54)	278/481 (58)

For peer review only

ETHICS AND DISSEMINATION

Prospective participants will be fully informed about the potential risks and benefits of participation and written informed consent will be obtained prior to recruitment. The study is being conducted according to protocols approved by the following ethics committees: Lao PDR Ministry of Health National Ethics Committee for Health Research (057/2013 NECHR), Oxford Tropical Research Ethics Committee (1050-13), Mongolian National Ethics Committee for Health Research, the WHO Regional Office for the Western Pacific (WPRO) Ethics Review Committee (2013.30.LAO.2.EPI), PNG IMR Institutional Review Board (1510), Government of PNG Medical Research Advisory Committee (15.18), and the Royal Children's Hospital/MCRI Human Research Ethics Committee (33177B, 33203E).

We plan on disseminating results to relevant stakeholders within Lao PDR, PNG and Mongolia, as well as submitting our findings for publication in relevant peer-reviewed journals and conferences.

DISCUSSION

The ability for low- and middle-income countries to monitor the indirect effects of PCV is critical. Maximising indirect effects is important because these effects comprise a substantial proportion of overall PCV impact and increase the cost-effectiveness of the vaccine.[39] Indirect protection is particularly important for individuals who are unable to be vaccinated or who have poor vaccine responses, such as infants too young to be vaccinated and the elderly. In addition, the Bill & Melinda Gates Foundation are supporting studies looking into the effectiveness of reduced dose schedules (1+1). This reduced dose schedule needs to maintain indirect effects following PCV introduction and this surveillance method provides a mechanism to determine when herd protection has been

For peer review only - http://bmjopen.bmj.com/site/about/guidelines.xhtml

achieved and whether it is maintained.[40] Furthermore, the ability to determine whether substantial herd immunity has been achieved may help to identify settings which are appropriate for introduction of reduced dose or modified schedules.

The results of our study will address the scarcity of literature determining the PCV coverage required to achieve substantial indirect effects, especially in low- and middle-income countries. The inclusion of three sites, which have contrasting vaccine schedules, baseline intensities of pneumococcal carriage and health-care systems, will also enable us to determine whether this vaccine coverage threshold differs by site and explore factors which may modify the vaccine coverage required to achieve indirect effects.

In this proposal, we describe a novel application of an analysis method to measure indirect effects. Although the isolation of a particular pneumococcal serotype from the nasopharynx of a child with ARI is not necessarily indicative of the serotype causing pneumococcal disease, reduced detection of VT pneumococci is likely to reflect reductions in disease due to VT pneumococci. Furthermore, our proposed analysis methods enable estimation of indirect effects in the absence of baseline pre-PCV data. This will be relevant for many low- and middle-income countries that have little or no baseline data and are considering options for surveillance to accompany the introduction of PCV.

Another key strength of our methods is the use of consistent molecular serotyping microarray methods across all three sites, enabling sensitive detection of multiple serotype carriage and ascertainment of serotype-specific density.[29] Conventional pneumococcal carriage studies typically detect the presence of a single serotype and may overlook the presence of vaccine serotypes occurring at lower densities. The results of this study will also add to the limited literature on the effects of PCV on vaccine-type carriage density, which may affect likelihood of transmission. [41]

Our proposed methods have several potential limitations. Firstly, there are significant challenges in determining accurate estimates of PCV coverage in resource-limited settings. In Mongolia and Lao PDR, where we are using administrative data, our estimates rely on the availability of accurate data about vaccine doses administered (numerator) as well as population estimates for the target age group (denominator). In both settings, we are conducting audits to assess the reliability of numerator data. Regarding denominator data, we are fortunate that a recent population census was conducted in Lao PDR in March 2015. However, we will be auditing denominator data in Mongolia, where population estimates are affected by large seasonal population movements between rural and urban settings. In PNG, the validity of the community surveys depends on representative sampling. However survey participation can vary and is affected by season (related to farming practices), as well as community trust and understanding of the study. To maximise participation, we conducted mobile health clinics alongside the community surveys.

The second main limitation relates to detecting pneumococcal carriage in our study population of children hospitalised with respiratory infection. In this population, carriage detection may be affected by the prior use of antibiotics.[42 43] To address this, we have, where possible, aimed to recruit patients

For peer review only - http://bmjopen.bmj.com/site/about/guidelines.xhtml

at admission, prior to receiving antibiotics or documented prior antibiotic use, which will be taken into account during analysis.

Thirdly, there are variations in methods and setting across the three sites. This limitation may impact on the comparability of our results across the three sites. The reason for the differences in methods between sites is that our study is built on pre-existing studies (pneumonia aetiology studies in Lao PDR and PNG, and a vaccine impact study in Mongolia) with established protocols.

To conclude, the results of this study will provide important feedback to national policy makers about the effects of newly introduced PCV programs in Lao PDR, Mongolia, and PNG. The results will help us understand the determinants of indirect effects and therefore guide strategies to maximise them. In particular, the results will also inform global policy about the vaccine coverage required to achieve substantial indirect effects in settings with different epidemiological characteristics of pneumococcal disease and carriage and will maximise the cost-effectiveness of the vaccine programs.

Acknowledgements:

We would like to acknowledge the Ministries of Health of Lao PDR and Mongolia, LOMWRU, PNG IMR and WHO and support from the Victorian Government's Operational Infrastructure Support Program. We would also like to thank study staff, laboratory staff and participating families.

The PneuCAPTIVE protocol development group includes the authors of the paper listed in the by-line and the following: Dashtseren Luvsantseren (NCCD, Ulaanbaatar, Mongolia), Bujinlkham (NCCD, Ulaanbaatar, Mongolia), Mukhchuluun Ulziibayar (NCCD, Ulaanbaatar, Mongolia), Dashpagam (NCCD, Ulaanbaatar, Mongolia), Audrey Dubot-Pérès (LOMHWRU, Vientiane, Lao PDR), Keodomphone Vilavong (LOMHWRU, Vientiane, Lao PDR), Anisone Chanthongthip (LOMHWRU, Vientiane, Lao PDR), Syladeth Chanthaphone (LOMHWRU, Vientiane, Lao PDR), Joycelyn Sapura (PNG IMR, Goroka, PNG), John Kave (PNG IMR, Goroka, PNG), Tonny Kumani (PNG IMR, Goroka, PNG), Wendy Kirarock (PNG IMR, Goroka, PNG).

Author's contributions: FR conceived the idea and designed the study. JL, SD, KF, PN, RL, RP, AX, DD and FR supported the development of country-specific protocols and study implementation in Lao PDR. CCB, RF, DL, WP and FR supported the development of country-specific protocols and study implementation in PNG. TM, SLV, CVM, KM, JC and FR supported the development of country-specific protocols and study implementation in Mongolia. CS, ED and JH devised the microbiological approach and laboratory protocols. JC, CN, RA, and FR devised the analysis plan. JC and FR drafted the manuscript. All authors provided feedback to the draft manuscript and have read and approved the final version.

Funding statement: This work is supported by the Bill & Melinda Gates Foundation grant number (OPP1115490). JC is completing a PhD at The University of Melbourne, funded by an Australian Government Research Training Program scholarship.

Competing interests: None declared.

For peer review only - http://bmjopen.bmj.com/site/about/guidelines.xhtml

REFERENCES

1. Wang H, Bhutta ZA, Coates MM, et al. Global, regional, national, and selected subnational levels of stillbirths, neonatal, infant, and under-5 mortality 1980-2015: a systematic analysis for the Global Burden of Disease Study 2015. *The Lancet* 2016;388(10053):1725-74. doi: 10.1016/S0140-6736(16)31575-6

2. Hill PC, Townend J, Antonio M, et al. Transmission of Streptococcus pneumoniae in rural Gambian villages: a longitudinal study. *Clinical Infectious Diseases* 2010;50(11):1468-76. doi: http://dx.doi.org/10.1086/652443

3. Hussain M, Melegaro A, Pebody RG, et al. A longitudinal household study of Streptococcus pneumoniae nasopharyngeal carriage in a UK setting. *Epidemiology and Infection* 2005;133(5):891-98. doi: http://dx.doi.org/10.1017/S0950268805004012

4. Gray BM, Converse GM, 3rd, Dillon HC, Jr. Epidemiologic studies of Streptococcus pneumoniae in infants: acquisition, carriage, and infection during the first 24 months of life. *The Journal of infectious diseases* 1980;142(6):923-33. [published Online First: 1980/12/01]

5. Varon E, Cohen R, Bechet S, et al. Invasive disease potential of pneumococci before and after the 13-valent pneumococcal conjugate vaccine implementation in children. *Vaccine* 2015;33(46):6178-85.

6. Johnson HL, Deloria-Knoll M, Levine OS, et al. Systematic evaluation of serotypes causing invasive pneumococcal disease among children under five: the pneumococcal global serotype project. *PLoS Med* 2010;7(10) doi: 10.1371/journal.pmed.1000348 [published Online First: 2010/10/20]

7. Loo JD, Conklin L, Fleming-Dutra KE, et al. Systematic review of the effect of pneumococcal conjugate vaccine dosing schedules on prevention of pneumonia. *The Pediatric infectious disease journal* 2014;33 Suppl 2:S140-51. doi: 10.1097/inf.0000000000000082 [published Online First: 2013/12/18]

8. Conklin L, Loo JD, Kirk J, et al. Systematic review of the effect of pneumococcal conjugate vaccine dosing schedules on vaccine-type invasive pneumococcal disease among young children. *The Pediatric infectious disease journal* 2014;33 Suppl 2:S109-18. doi: 10.1097/inf.0000000000000078 [published Online First: 2013/12/18]

9. Le Saux N. Pneumococcal conjugate vaccines for preventing otitis media. *Paediatrics & Child Health* 2016;21(2):89-90.

10. Loo JD, Conklin L, Fleming-Dutra KE, et al. Systematic review of the indirect effect of pneumococcal conjugate vaccine dosing schedules on pneumococcal disease and colonization. *Pediatric Infectious Disease Journal* 2014;33 Suppl 2:S161-71.

11. Direct and indirect effects of routine vaccination of children with 7-valent pneumococcal conjugate vaccine on incidence of invasive pneumococcal disease--United States, 1998-2003. *MMWR Morbidity and mortality weekly report* 2005;54(36):893-7. [published Online First: 2005/09/16]

12. Tsaban G, Ben-Shimol S. Indirect (herd) protection, following pneumococcal conjugated vaccines introduction: A systematic review of the literature. *Vaccine* 2017;35(22):2882-91. doi: 10.1016/j.vaccine.2017.04.032 [published Online First: 2017/04/30]

13. Loughlin AM, Hsu K, Silverio AL, et al. Direct and indirect effects of PCV13 on nasopharyngeal carriage of PCV13 unique pneumococcal serotypes in Massachusetts' children. *The Pediatric infectious disease journal* 2014;33(5):504-10. doi: 10.1097/inf.0000000000000279 [published Online First: 2014/03/29]

14. Grant LR, Hammitt LL, O'Brien SE, et al. Impact of the 13-valent pneumococcal conjugate vaccine on pneumococcal carriage among American Indians. *The Pediatric infectious disease journal* 2016;35(8):907-14. doi: 10.1097/inf.0000000000001207 [published Online First: 2016/05/14]

For peer review only - http://bmjopen.bmj.com/site/about/guidelines.xhtml

15. International Vaccine Access Center - John Hopkins Bloomberg School of Public Health. VIEW-hub 2016 [accessed 17/12/2016.

16. Shiri T, Datta S, Madan J, et al. Indirect effects of childhood pneumococcal conjugate vaccination on invasive pneumococcal disease: a systematic review and meta-analysis. *The Lancet Global Health* 2017;5(1):e51-e59. doi: 10.1016/s2214-109x(16)30306-0

17. Jayasinghe S, Menzies R, Chiu C, et al. Long-term impact of a “3+0” schedule for 7 and 13 valent pneumococcal conjugate vaccines on invasive pneumococcal disease in Australia, 2002-2014. *Clinical Infectious Diseases* 2016 doi: 10.1093/cid/ciw720

18. Mackenzie GA, Hill PC, Jeffries DJ, et al. Effect of the introduction of pneumococcal conjugate vaccination on invasive pneumococcal disease in The Gambia: a population-based surveillance study. *The Lancet Infectious diseases* 2016;16(6):703-11. doi: 10.1016/s1473-3099(16)00054-2 [published Online First: 2016/02/22]

19. von Gottberg A, de Gouveia L, Tempia S, et al. Effects of vaccination on invasive pneumococcal disease in South Africa. *The New England journal of medicine* 2014;371(20):1889-99. doi: 10.1056/NEJMoa1401914 [published Online First: 2014/11/12]

20. Rodgers GL, Klugman KP. Surveillance of the impact of pneumococcal conjugate vaccines in developing countries. *Human Vaccines and Immunotherapeutics* 2016;12(2):417-20. doi: http://dx.doi.org/10.1080/21645515.2015.1057671

21. Gladstone RA, Jefferies JM, Tocheva AS, et al. Five winters of pneumococcal serotype replacement in UK carriage following PCV introduction. *Vaccine* 2015;33(17):2015-21.

22. Dunne EM, Manning J, Russell FM, et al. Effect of pneumococcal vaccination on nasopharyngeal carriage of Streptococcus pneumoniae, Haemophilus influenzae, Moraxella catarrhalis, and Staphylococcus aureus in Fijian children. *J Clin Microbiol* 2012;50(3):1034-8. doi: 10.1128/jcm.06589-11 [published Online First: 2011/12/16]

23. Mosser JF, Grant LR, Millar EV, et al. Nasopharyngeal carriage and transmission of Streptococcus pneumoniae in American Indian households after a decade of pneumococcal conjugate vaccine use.[Erratum appears in PLoS One. 2014;9(3):e93878]. *PLoS ONE [Electronic Resource]* 2014;9(1):e79578.

24. Lehmann D, Gratten M, Montgomery J. Susceptibility of pneumococcal carriage isolates to penicillin provides a conservative estimate of susceptibility of invasive pneumococci. *The Pediatric infectious disease journal* 1997;16(3):297-305. [published Online First: 1997/03/01]

25. Nguyen VH, Dubot-Peres A, Russell FM, et al. Acute respiratory infections in hospitalized children in Vientiane, Lao PDR - the importance of Respiratory Syncytial Virus. *Scientific reports* 2017;7(1):9318. doi: 10.1038/s41598-017-09006-6 [published Online First: 2017/08/26]

26. Blyth CC, Ford R, Sapura J, et al. Childhood pneumonia and meningitis in the Eastern Highlands Province, Papua New Guinea in the era of conjugate vaccines: study methods and challenges. *Pneumonia (Nathan Qld)* 2017;9:5. doi: 10.1186/s41479-017-0029-y [published Online First: 2017/07/14]

27. Satzke C, Turner P, Virolainen-Julkunen A, et al. Standard method for detecting upper respiratory carriage of Streptococcus pneumoniae: updated recommendations from the World Health Organization Pneumococcal Carriage Working Group. *Vaccine* 2013;32(1):165-79. doi: 10.1016/j.vaccine.2013.08.062 [published Online First: 2013/12/18]

28. Dunne EM, Manning J, Russell FM, et al. Effect of Pneumococcal Vaccination on Nasopharyngeal Carriage of Streptococcus pneumoniae, Haemophilus influenzae, Moraxella catarrhalis, and Staphylococcus aureus in Fijian Children. *Journal of Clinical Microbiology* 2012;50(3):1034-38. doi: 10.1128/JCM.06589-11

For peer review only - http://bmjopen.bmj.com/site/about/guidelines.xhtml

For peer review only

29. Satzke C, Dunne EM, Porter BD, et al. The PneuCarriage Project: A Multi-Centre Comparative Study to Identify the Best Serotyping Methods for Examining Pneumococcal Carriage in Vaccine Evaluation Studies. *PLoS Medicine / Public Library of Science* 2015;12(11):e1001903; discussion e03.

30. Newton R, Hinds J, Wernisch L. Empirical Bayesian models for analysing molecular serotyping microarrays. *BMC bioinformatics* 2011;12:88. doi: 10.1186/1471-2105-12-88 [published Online First: 2011/04/02]

31. Scott P, Rutjes AW, Bermetz L, et al. Comparing pneumococcal conjugate vaccine schedules based on 3 and 2 primary doses: systematic review and meta-analysis. *Vaccine* 2011;29(52):9711-21. doi: 10.1016/j.vaccine.2011.07.042 [published Online First: 2011/08/09]

32. Chan J. High agreement between the new Mongolian electronic immunisation register and clinic immunisation records: a health centre based audit. *Western Pacific Surveillance and Response Journal* 2017;In press

33. Deen J, Ali M, Sack D. Methods to assess the impact of mass oral cholera vaccination campaigns under real field conditions. *PLoS ONE* 2014;9(2):e88139. doi: 10.1371/journal.pone.0088139

34. Khatib AM, Ali M, von Seidlein L, et al. Effectiveness of an oral cholera vaccine in Zanzibar: findings from a mass vaccination campaign and observational cohort study. *The Lancet Infectious diseases* 2012;12(11):837-44. doi: 10.1016/s1473-3099(12)70196-2 [published Online First: 2012/09/08]

35. Moore MR, Hyde TB, Hennessy TW, et al. Impact of a conjugate vaccine on community-wide carriage of nonsusceptible Streptococcus pneumoniae in Alaska. *Journal of Infectious Diseases* 2004;190(11):2031-8.

36. Usuf E, Bottomley C, Adegbola RA, et al. Pneumococcal carriage in sub-Saharan Africa - A systematic review. *PLoS ONE* 2014;9 (1) (no pagination)(e85001) doi: http://dx.doi.org/10.1371/journal.pone.0085001

37. Aho C, Michael A, Yoannes M, et al. Limited impact of neonatal or early infant schedules of 7-valent pneumococcal conjugate vaccination on nasopharyngeal carriage of Streptococcus pneumoniae in Papua New Guinean children: A randomized controlled trial. *Vaccine Reports* 2016;6:36-43. doi: http://dx.doi.org/10.1016/j.vacrep.2016.08.002

38. Sterne JAC, White IR, Carlin JB, et al. Multiple imputation for missing data in epidemiological and clinical research: potential and pitfalls. *Bmj* 2009;338 doi: 10.1136/bmj.b2393

39. Isaacman DJ, Strutton DR, Kalpas EA, et al. The impact of indirect (herd) protection on the cost-effectiveness of pneumococcal conjugate vaccine. *Clinical therapeutics* 2008;30(2):341-57. doi: 10.1016/j.clinthera.2008.02.003 [published Online First: 2008/03/18]

40. Rodgers GL, Klugman KP. A new paradigm in pneumococcal conjugate vaccination: moving from individual to herd protection. *International journal of infectious diseases : IJID : official publication of the International Society for Infectious Diseases* 2017 doi: 10.1016/j.ijid.2017.04.015

41. Roca A, Bottomley C, Hill PC, et al. Effect of age and vaccination with a pneumococcal conjugate vaccine on the density of pneumococcal nasopharyngeal carriage. *Clinical Infectious Diseases* 2012;55(6):816-24.

42. Abdullahi O, Karani A, Tigoi CC, et al. The prevalence and risk factors for pneumococcal colonization of the nasopharynx among children in Kilifi District, Kenya. *PLoS One* 2012;7(2):e30787. doi: 10.1371/journal.pone.0030787 [published Online First: 2012/03/01]

43. Alpkvist H, Athlin S, Naucler P, et al. Clinical and Microbiological Factors Associated with High Nasopharyngeal Pneumococcal Density in Patients with Pneumococcal Pneumonia. *PLoS One* 2015;10(10):e0140112. doi: 10.1371/journal.pone.0140112 [published Online First: 2015/10/16]

For peer review only - http://bmjopen.bmj.com/site/about/guidelines.xhtml

For peer review only

For peer review only - http://bmjopen.bmj.com/site/about/guidelines.xhtml

Determining the pneumococcal conjugate vaccine coverage required for indirect protection against vaccine-type pneumococcal carriage in low- and middle-income countries: a protocol for a prospective observational study

Journal:	*BMJ Open*
Manuscript ID	bmjopen-2018-021512.R2
Article Type:	Protocol
Date Submitted by the Author:	29-Mar-2018

Complete List of Authors:	Chan, Jocelyn; Murdoch Childrens Research Institute, Pneumococcal Research Group; The University of Melbourne, Department of Paediatrics<br>Nguyen, Cattram; Murdoch Childrens Research Institute, Pneumococcal Research; The University of Melbourne, Department of Paediatrics<br>Lai, Jana; Murdoch Childrens Research Institute, Pneumococcal Research Group<br>Dunne, Eileen; Murdoch Childrens Research Institute, Pneumococcal Research Group; The University of Melbourne, Department of Paediatrics<br>Andrews, Ross; Menzies School of Health Research, Charles Darwin University, Global & Tropical Health Division; Australian National University, National Centre for Epidemiology & Population Health<br>Blyth, Christopher C.; University of Western Australia, School of Medicine; Princess Margaret Hospital for Children, Department of Infectious Diseases<br>Datta, Siddhartha; World Health Organization<br>Fox, Kim; World Health Organization, Regional Office for the Western Pacific<br>Ford, Rebecca ; Papua New Guinea Institute of Medical Research<br>Hinds, Jason; St George's- University of London, Institute for Infection and Immunity; London Bioscience Innovation Centre, BUGS Bioscience<br>La Vincente, Sophie; Murdoch Childrens Research Institute, Pneumococcal Research Group<br>Lehmann, Deborah; Telethon Kids Institute, University of Western Australia, Wesfarmers Centre for Vaccines and Infectious Diseases<br>Lim, Ruth; Murdoch Childrens Research Institute, Pneumococcal Research Group<br>Mungun, Tuya ; Ministry of Health, National Center of Communicable Diseases (NCCD)<br>Newton, Paul ; Lao-Oxford-Mahosot Hospital-Wellcome Trust Research Unit , Microbiology Laboratory, Mahosot Hospital; University of Oxford, Centre for Tropical Medicine and Global Health<br>Phetsouvanh, Rattanaphone; Lao-Oxford-Mahosot Hospital-Wellcome Trust Research Unit, Microbiology Laboratory, Mahosot Hospital; University of Oxford, Centre for Tropical Medicine and Global Health<br>Pomat, Willie; PNG Institute of Medical Research, Infection and Immunity; Telethon Kids Institute, University of Western Australia, Wesfarmers Centre for Vaccines and Infectious Diseases<br>Xeuatvongsa, Anonh; Ministry of Health, National Immunization Programme

	von Mollendorf, Claire; Murdoch Childrens Research Institute, Pneumococcal Research Group; The University of Melbourne, Department of Paediatrics<br>Dance, David; Lao-Oxford-Mahosot Hospital-Wellcome Trust Research Unit, Microbiology Laboratory, Mahosot Hospital; University of Oxford, Centre for Tropical Medicine and Global Health<br>Satzke, Catherine; Murdoch Childrens Research Institute, Pneumococcal Research; The University of Melbourne at the Peter Doherty Institute for Infection and Immunity, Department of Microbiology and Immunology<br>Muholland , Kim; Murdoch Childrens Research Institute, Pneumococcal Research Group; London School of Hygiene and Tropical Medicine, Department of Infectious Disease Epidemiology<br>Russell, F. M.; Murdoch Childrens Research Institute, Pneumococcal Research Group; The University of Melbourne, Centre for International Child Health, Department of Paediatrics
**Primary Subject Heading**:	Global health
Secondary Subject Heading:	Infectious diseases, Epidemiology
Keywords:	Public health < INFECTIOUS DISEASES, Paediatric infectious disease & immunisation < PAEDIATRICS, Respiratory infections < THORACIC MEDICINE

SCHOLARONE™
Manuscripts

For peer review only

DETERMINING THE PNEUMOCOCCAL CONJUGATE VACCINE COVERAGE REQUIRED FOR INDIRECT PROTECTION AGAINST VACCINE-TYPE PNEUMOCOCCAL CARRIAGE IN LOW- AND MIDDLE-INCOME COUNTRIES: A PROTOCOL FOR A PROSPECTIVE OBSERVATIONAL STUDY

Authors:

Chan J[1,2], Nguyen CD[1,2], Lai JYR[1], Dunne EM[1,2], Andrews R[3,4], Blyth CC[5,6], Datta S[7], Fox K[8], Ford R[9], Hinds J[10,11], La Vincente S[1] , Lehmann D[12], Lim R[1], Mungun T[13], Newton PN[14,15], Phetsouvanh R[14,15*], Pomat W[9,12], Xeuatvongsa A[16], von Mollendorf C[1,2], Dance DAB[14,15] Satzke C[1,17], Mulholland K[1,18], Russell FM[1,19] for the PneuCAPTIVE Protocol Group

1. Pneumococcal Research Group, Murdoch Children's Research Institute, Melbourne, Australia.
2. Department of Paediatrics, The University of Melbourne, Melbourne, Australia.
3. Global & Tropical Health Division, Menzies School of Health Research, Charles Darwin University, Darwin, Australia.
4. National Centre for Epidemiology & Population Health, Australian National University, Canberra, Australia
5. University of Western Australia, School of Medicine, Perth, Australia;
6. Department of Infectious Diseases, Princess Margaret Hospital, Perth, Australia;
7. World Health Organization, Vientiane, Lao People's Democratic Republic
8. Regional Office for the Western Pacific, World Health Organization, Manila, Philippines.
9. Papua New Guinea Institute of Medical Research, Infection and Immunity Unit, Goroka, Papua New Guinea.
10. Institute for Infection and Immunity, St George's- University of London, London, United Kingdom.
11. BUGS Bioscience, London Bioscience Innovation Centre, London, United Kingdom.
12. Wesfarmers Centre for Vaccines and Infectious Diseases, Telethon Kids Institute, University of Western Australia, Perth, Australia.
13. National Center of Communicable Diseases (NCCD), Ministry of Health, Ulaanbaatar, Mongolia.
14. Lao-Oxford-Mahosot Hospital-Wellcome Trust Research Unit (LOMHWRU), Microbiology Laboratory, Mahosot Hospital, Vientiane, Lao PDR.
15. Centre for Tropical Medicine and Global Health, University of Oxford, Oxford, United Kingdom.
16. National Immunization Programme, Ministry of Health, Vientiane, Lao PDR.
17. Department of Microbiology and Immunology, The University of Melbourne at the Peter Doherty Institute for Infection and Immunity, Melbourne, Australia
18. Department of Infectious Disease Epidemiology, London School of Hygiene and Tropical Medicine, London, United Kingdom.

For peer review only - http://bmjopen.bmj.com/site/about/guidelines.xhtml

19. Centre for International Child Health, Department of Paediatrics, The University of Melbourne, Melbourne, Australia.

*Deceased

Corresponding author: Jocelyn Chan

Pneumococcal Research Group,
Murdoch Children's Research Institute
50 Flemington Road, Parkville 3052 VIC
Australia

Email: jocelyn.chan@mcri.edu.au

Telephone: +61 3 9345 4968

For peer review only

For peer review only - http://bmjopen.bmj.com/site/about/guidelines.xhtml

ABSTRACT

Introduction

Pneumococcal conjugate vaccines (PCVs) prevent disease through both direct protection of vaccinated individuals, and indirect protection of unvaccinated individuals by reducing nasopharyngeal (NP) carriage and transmission of vaccine-type (VT) pneumococci. While the indirect effects of PCV vaccination are well described, the PCV coverage required to achieve the indirect effects is unknown. We will investigate the relationship between PCV coverage and vaccine-type carriage among under-vaccinated children using hospital-based NP pneumococcal carriage surveillance at three sites in the Asia-Pacific region.

Methods and analysis

We are recruiting cases, defined as children aged 2-59 months admitted to participating hospitals with acute respiratory infection in Lao People's Democratic Republic, Mongolia and Papua New Guinea. Thirteen-valent PCV (PCV13) status is obtained from written records. NP swabs are collected according to standard methods, screened using *lytA* qPCR and serotyped by microarray. Village-level vaccination coverage, for the resident communities of the recruited cases, is determined using administrative data or community survey. Our analysis will investigate the relationship between VT carriage among under-vaccinated cases (indirect effects) and vaccine coverage using generalised estimating equations.

Ethics and dissemination

Ethical approval has been obtained from the relevant ethics committees at participating sites. The results are intended for publication in open-access peer-reviewed journals and will demonstrate methods suitable for low- and middle-income countries to monitor vaccine impact, and inform vaccine policy makers about the PCV coverage required to achieve indirect protection.

STRENGTHS AND LIMITATIONS

- We describe a novel application of study methods that enable monitoring the indirect effects of pneumococcal conjugate vaccines (PVCs) using pneumococcal carriage and in the absence of baseline data
- The methods do not measure indirect protection against disease. However, a reduction in VT carriage among undervaccinated cases suggests likely reductions in VT disease, since carriage is a precursor for disease The use of molecular serotyping microarray enables detection of multiple serotype carriage and serotype-specific carriage density
- The inclusion of three sites, which have contrasting vaccine schedules and pneumococcal epidemiology, will enable us to explore factors which may modify the vaccine coverage required to achieve indirect effects however variations in methods and settings may impact on the comparability of our results across the three sites

For peer review only - http://bmjopen.bmj.com/site/about/guidelines.xhtml

INTRODUCTION

Infections due to *Streptococcus pneumoniae* (the pneumococcus), including pneumonia, meningitis and septicaemia, are a leading cause of morbidity and mortality among children and elderly, especially in low- and middle-income countries.[1] The main reservoir for the pneumococcus is the human nasopharynx. Pneumococcal carriage peaks in young children, an important group for transmission of pneumococci to older age groups,[2 3] and is a precursor for disease.[4] There are over 90 known serotypes of pneumococci, with differing capacities for causing disease.[5]

The introduction of the pneumococcal conjugate vaccine (PCV) has resulted in substantial reductions in pneumococcal disease in many settings.[6] These reductions are mediated by direct effects on vaccinated children as well as indirect effects on under-vaccinated children and adults through a reduction in transmission and subsequent nasopharyngeal (NP) carriage of pneumococcal serotypes included in the vaccine.[7-10] The indirect effects account for a substantial component of the overall vaccine effect. Following the introduction of the 7-valent PCV (PCV7) into the routine vaccination program in the USA, twice as many invasive cases were prevented through indirect effects compared to direct effects.[11]

The magnitude of indirect effects following introduction of PCV varies considerably by setting. Reductions in invasive pneumococcal disease (IPD) among adults ranged from 8.8% among adults in Denmark three years after PCV implementation, to 70% reduction among adults in Taiwan, seven years after PCV implementation.[12] A review of the literature found that higher vaccine coverage, higher baseline rates of pneumococcal disease and greater time elapsed since PCV introduction were all associated with greater degrees of indirect effects.[12] However, the majority of these studies were conducted in high-income country settings using a similar vaccine schedule, with relatively high levels of vaccine coverage.

The threshold of vaccination coverage required to achieve significant indirect PCV effects on either pneumococcal carriage or disease outcomes are not well understood. Studies of NP carriage indicate that there is variability in the coverage required to achieve significant indirect effects. Two studies in the US examined this question for the 13-valent PCV (PCV13) that superseded 7-valent (PCV7) immunisation. A vaccine coverage of 58% among American Indian children in south-western USA and 75% coverage among children in Massachusetts resulted in a 50% decline in the prevalence of carriage of six PCV13 serotypes (i.e. PCV13 types not included in PCV7) carried by under-vaccinated children.[13 14] The US uses a 3+1 schedule (three primary doses with a booster) and had a catch-up program up to five years of age. Many low- and middle-income countries utilise a 3+0 schedule. [15] More data are needed from a range of low- and middle-income countries to determine the vaccination coverage required to achieve indirect protection from PCVs, and to determine factors which may modify the vaccine coverage required for indirect effects, such as baseline carriage prevalence, indicating intensity of transmission, vaccine schedules and use of catch-up campaigns.

Existing systematic reviews indicate that vaccine schedules do not impact on the degree of indirect effects on IPD, however the studies included in these reviews predominantly use a 3+1 schedule or a

For peer review only - http://bmjopen.bmj.com/site/about/guidelines.xhtml

2+1 schedule (two primary doses with a booster) and have limited studies using PCV13.[12 16] Few studies using the 3+0 schedule (three primary doses with no booster) are available in the published literature, despite the 3+0 schedule being widely used, especially in low- and middle-income countries. A recent study from Australia, the only high-income country to use the 3+0 schedule, concluded that the booster dose may be important for obtaining indirect protection because of the lower indirect effects observed compared to other high-income country settings.[17]

Despite the gradual introduction of PCVs in low- and middle-income countries over the last decade, there have been few studies published on the impacts of PCV in these settings.[18 19] This is because the predominant method for assessing vaccine impact, IPD surveillance, is costly and resource-intensive to establish – requiring laboratory capacity as well as the collection of large numbers of samples, obtained using aseptic techniques, in order to detect a relatively rare outcome.[20] Furthermore, baseline data prior to vaccine introduction required for impact evaluations are often not available in these settings.

In the absence of IPD surveillance, low- and middle-income countries require a method to evaluate direct and indirect vaccine effects. We propose using NP carriage surveillance in children hospitalised with acute respiratory infection to monitor the indirect effects of PCV13 in the study called PneuCAPTIVE (PNEUmococcal CArriage in Pneumonia To Investigate Vaccine Effects).

In this context, we refer to "carriage" as the detection of pneumococci in the nasopharynx of a child with acute respiratory infection. A reduction in vaccine-type (VT) pneumococcal carriage suggestslikely reductions in disease due to VT pneumococci, since carriage is a precursor to disease.[4] Existing studies among healthy children have demonstrated reductions in VT carriage following PCV introduction, while overall carriage remains stable due to replacement with non-vaccine type (NVT) carriage.[21 22]

We aim to determine the PCV13 coverage required to demonstrate substantial indirect effects of PCV using NP carriage surveillance in children hospitalised with acute respiratory infection (ARI) in three settings within the Asia-Pacific region. We are focussing on children under five years of age because studies have shown that children are the main reservoir for pneumococci, and reductions in transmission within this age group are likely to result in reductions in VT carriage among older age groups. [2 3 23]

As this is a novel method for determining indirect effects, we will also aim to determine whether changes in patterns of VT and NVT pneumococcal carriage among children with ARI are reflective of changes in serotypes circulating in the community, noting that carriage in our cohort may be more reflective of serotypes causing disease. [5 24]

OBJECTIVES

Our objectives are to: (1) investigate the relationship between PCV13 coverage and VT carriage among under-vaccinated cases, defined as children (indirect effects) aged 2-59 months with an ARI in Lao People's Democratic Republic (Lao PDR), Mongolia and Papua New Guinea (PNG); (2) describe

For peer review only - http://bmjopen.bmj.com/site/about/guidelines.xhtml

monthly trends in VT carriage prevalence among cases and contacts. Contacts are defined as children 0-59 months of age, who have slept in the same house as or played with the case during the preceding three weeks; (3) investigate the relationship between PCV13 coverage and VT carriage among under-vaccinated contacts (indirect effects) and caregivers living in the community; and (4) compare the PCV13 coverage required to demonstrate indirect effects of PCV13 by site and determine the degree to which site-specific factors, such as pneumococcal carriage rates and densities, vaccine schedule and use of catch-up campaigns, account for differences in the PCV13 coverage required to demonstrate indirect effects.

METHODS

Study design

We are conducting prospective hospital-based observational studies in Lao PDR, Mongolia and PNG. We are recruiting children 2-59 months of age presenting with ARI and obtaining NP swabs to determine prevalence and density of VT carriage. We are determining the PCV13 status of each case using written record. Recruitment will occur over at least three years and up to five years post-PCV13 introduction at each site.

Concurrently, we are determining vaccination coverage at the resident village or subdistrict of each recruited case, using either administrative data or vaccination coverage surveys.

Study settings

Lao PDR site

The Lao PDR PneuCAPTIVE study is embedded within a hospital-based study of ARI aetiology, in collaboration with Lao Oxford Mahosot Hospital-Wellcome Trust Research Unit (LOMWRU), the World Health Organization (WHO) and the Lao PDR Ministry of Health [25]. PCV13 was introduced in October 2013, using a 3+0 schedule at 6, 10 and 14 weeks of age and a catch-up program up to 12 months of age (Table 1). We are recruiting cases at Mahosot Hospital, one of the largest paediatric referral hospitals in Vientiane, the capital of Lao PDR. Recruitment started in December 2013.

Mongolia site

The Mongolian PneuCAPTIVE study is embedded within a hospital-based paediatric pneumonia surveillance to determine vaccine impact which is conducted in partnership between Murdoch Children's Research Institute (MCRI), WHO and the Mongolian Ministry of Health. PCV13 was introduced in June 2016, using a modified 2+1 schedule at 2, 4 and 9 months of age, within the two 'Phase 1' districts within Ulaanbaatar, the capital of Mongolia as part of a phased introduction (Table 1), with a catch-up program of two doses one month apart for those up to 24 months of age. We are recruiting cases, residing in the two phase 1 districts, at the two district hospitals and the tertiary referral paediatric hospital for Mongolia, the Maternal and Child Hospital (MCH). Sampling started November 2015.

PNG site

The PNG PneuCAPTIVE study represents a collaboration between the PNG Institute of Medical Research (IMR), Telethon Kids Institute, the University of Western Australia and MCRI. It is an

For peer review only - http://bmjopen.bmj.com/site/about/guidelines.xhtml

extension of a pneumonia aetiology study that commenced in 2013 [26]. PCV13 was introduced to PNG in October 2014 using a 3+0 schedule at 1, 2 and 3 months of age (Table 1), however was not widely distributed in the Eastern Highlands Province until late 2015. We are recruiting cases at the Eastern Highlands Provincial Hospital, the major referral hospital for the Eastern Highland Province, as well as nearby clinics in Goroka, the capital of the Eastern Highlands Province. Recruitment started April 2016. In PNG, we are also recruiting caregivers, as well as contacts, defined as children 0-59 months of age, who have slept in the same house as or played with the case during the preceding three weeks. This will enable us to determine whether changes in patterns of VT pneumococcal carriage in the hospitalised cases are reflective of changes within the community, as well as to examine indirect effects in the adult age group.

Table 1: Key aspects of the 13-valent pneumococcal conjugate vaccination (PCV13) program by site, Lao People's Democratic Republic (PDR), Mongolia and Papua New Guinea (PNG)

	Lao PDR	Mongolia	PNG
Year of PCV13 introduction	Oct 2013	Jun 2016	Oct 2014
Location of PCV13 introduction	National	Two districts in Ulaanbaatar	National
PCV13 schedule	3+0 (6,10 and 14 weeks)	2+1 (2,4, and 9 months)	3+0 (1, 2 and 3 months)
Presence of a catch-up program	Catch-up of three doses up to 12 months of age	Catch-up of two doses two months apart up to 24 months of age	None

Case recruitment and data collection

Participant recruitment and data collection are consistent across the three sites; however there are some local adaptations to the protocol at each site which are summarised in Table 2. These adaptations are due to the PneuCAPTIVE study being nested within other existing studies, described above.

Cases are eligible for inclusion in the PneuCAPTIVE study if they are 2-59 months of age, and presenting with ARI (defined in Table 2 below). All cases with fever or respiratory symptoms are screened for inclusion. In Mongolia, we are restricting recruitment to patients living within the two 'Phase 1' districts which have commenced PCV13 in 2016. In PNG, we are restricting recruitment to patients living within one hour of the town as follow-up is logistically challenging.

Regarding recruitment, in Lao PDR, study staff are screening potential recruits from Monday to Friday each week. However, they are obtaining clinical information from medical records for all eligible cases, including those admitted at weekends to ensure we have a representative sample. In Mongolia, caregivers of all eligible children presenting to the hospital will be approached for recruitment as part of the larger PCV impact study. However, for the purposes of this study we will

For peer review only - http://bmjopen.bmj.com/site/about/guidelines.xhtml

select a random sample of 33 cases per month for microbiological testing. In PNG recruitment takes place 4-5 days per week.

Table 2: Patient eligibility by site, Lao People's Democratic Republic (PDR), Mongolia and Papua New Guinea (PNG)

**Site**	Lao PDR	Mongolia	PNG
**Inclusion criteria**	2-59 months of age and presenting with:		
**Definition of acute respiratory infection**	Fever (parent report or measured) AND one of cough OR dyspnoea OR rhinitis OR abnormal chest auscultation	Cough OR dyspnoea AND tachypnoea* OR hypoxia OR chest indrawing	Cough AND tachypnoea* AND lower chest wall indrawing
**Exclusion criteria**	-	Lives outside Phase 1 district<br>Admitted with pneumonia in the last 14 days	Lives ≥ 1 hour outside town OR hospitalisation in past 14 days
**Recruitment site**	Inpatient setting only	Inpatient setting only	Inpatient and outpatient setting
**Sampling**	Monday-Friday	A random sample (33 per month) of all enrolled cases are selected for testing	Monday- Friday
**Sampling period**	December 2013-November 2019	November 2015 – October 2018†	April 2016 – March 2019
**Study population**	Mahosot Hospital is one of two tertiary-level paediatric hospitals in Vientiane and receives a mix of patients from urban Vientiane city, rural Vientiane province and other provinces.	The two secondary-level district hospitals and a tertiary-level Maternal and Child Health hospital service the vast majority of children in the two districts that received PCV. There are a limited number of paediatric beds at private hospitals in Ulaanbaatar.	The Eastern Highlands Provincial hospital is the sole hospital for the province. Study population includes urban and rural households within 1 hour drive of Goroka.

* Tachypnoea is defined as greater than or equal to 50 breaths per minute

† A one-year extension (until June 2019) has been sought for the Mongolian site

For peer review only - http://bmjopen.bmj.com/site/about/guidelines.xhtml

After determining eligibility and obtaining informed parental consent, we complete a questionnaire to obtain: demographic data, clinical data, PCV13 status, and risk factors for vaccination and NP carriage, including prior antibiotic use (see analysis section below for complete list). Vaccination status is determined using written records – either parent-held immunisation records or health centre administrative records.

We then collect an NP swab according to WHO guidelines and store it in 1 ml skim milk tryptone glucose glycerol (STGG) medium. [27] Swabs are vortexed, aliquoted, and stored frozen at -80^{0}C within 8 hours of collection, and transported from all three sites to the Pneumococcal Research laboratory at MCRI on dry ice or in liquid nitrogen, where they will be stored at -80^{0}C.

Laboratory methods

All samples are screened for the presence of pneumococci using real-time quantitative PCR (qPCR) assay targeting the pneumococcal *lytA* gene.[28] Genomic DNA are extracted from 100 µl of STGG using a MagNA Pure LC Machine (Roche) using the DNA Isolation Kit III (Bacteria, Fungi) (Roche) following an enzymatic lysis treatment. The pneumococcal load is estimated by reference to a standard curve.

All swabs that are *lytA* positive or equivocal are molecular serotyped using BµG@S Senti-SP v1.5 microarray (BUGS Bioscience) as previously described. [29]

Serotype-specific pneumococcal density is calculated using the relative abundance of each serotype identified, as determined using microarray and interpreted with the assistance of a Bayesian random effects model as previously described,[29 30] and the overall pneumococcal load as determined by the *lytA* qPCR.

Key definitions

The primary outcome, VT carriage, is defined as the NP carriage of at least one pneumococcal serotype included in the PCV13 vaccine, i.e. serotypes 1, 3, 4, 5, 6A, 6B, 7F, 9V, 14, 18C, 19A, 19F, and 23F. In the context of multiple serotype carriage, VT carriage will be defined as the presence of at least one VT serotype regardless of the presence of other serotypes. A secondary outcome is VT carriage density (CFU/mL) which is defined as an aggregate of the serotype-specific density for each of the VTs carried by the case, and will be reported as a continuous variable using a log scale to account for large variations in density and a skewed distribution.

Vaccine history will be defined based on documented evidence of receiving an adequate number of PCV doses to provide a protective immune response against vaccine serotypes at least 14 days prior to study enrolment.[31] For children < 12 months of age, 'vaccinated' is defined as two or more PCV13 doses. For children 12 months of age or older, 'vaccinated' is defined as receipt of 2 doses in the first year of life or at least 1 dose after the age of 12 months. Conversely, a case will be defined as 'under-vaccinated' if they have received less than the adequate number of PCV doses (including those never vaccinated). Sensitivity analyses will be conducted using varying definitions of vaccinated including receiving at least one dose of vaccine at any age.

For peer review only - http://bmjopen.bmj.com/site/about/guidelines.xhtml

Vaccine coverage data collection

For each under-vaccinated case recruited (including those with and without VT pneumococci), we are determining their resident village or subdistrict vaccination coverage. In Mongolia and Lao PDR, we are using administrative data. The resident village or subdistrict is identified using relevant administrative codes, which are determined by local staff on enrolment. This determines the health centre's administrative boundary for the provision of immunisation services, whereas in PNG, we are conducting community surveys within 10 days of discharge in the village where the case is living (Table 3). We are surveying all children less than five years of age, from households within 10-20 minutes' walk of the case, since this is the group of children with whom the case is mostly likely to interact and therefore influence their carriage status.

To determine the reliability of our methods in Lao PDR, we plan on comparing our coverage estimates with a National Immunisation Survey, conducted according to WHO guidelines in 2015, when provincial level estimates from this survey become available. In Mongolia, we have validated the newly introduced electronic immunisation record against clinic health records, finding a high degree of concordance.[32] We are also in the process of validating the population registers at health centres in Mongolia.

Table 3: Vaccination coverage data by site, Lao People's Democratic Republic (PDR), Mongolia and Papua New Guinea

**Site**	Lao PDR	Mongolia	Papua New Guinea
**Source of numerator data**	Health centre records	Electronic immunisation record	Community surveys
**Source of denominator data**	Lao PDR Population and Housing Census 2015	Health centre population register*	

* All children are required to be registered at the health centre servicing their resident subdistrict in order to receive health services.

Data management

In Lao PDR and Mongolia, study staff are double-entering data using a RedCAP (Research Electronic Data Capture) and Microsoft Access (Microsoft Corporation) database, respectively. In PNG, data are checked by a monitor prior to being entered into Filemaker Pro (FileMaker Inc.). We are conducting regular double-entry discrepancy checks and logic checks using Stata Statistical Software (College Station, TX: StataCorp LLC).

Analysis

All analyses will be completed using Stata Statistical Software (College Station, TX: StataCorp LLC). We will summarise continuous variables using mean and standard deviation (or median and interquartile range for non-symmetrical data). Categorical variables will be summarised using frequency counts and percentages.

For peer review only - http://bmjopen.bmj.com/site/about/guidelines.xhtml

Objective 1: Relationship between vaccine coverage and indirect effects

We want to investigate the relationship between VT carriage and density among under-vaccinated cases (indirect effects) and subdistrict/village PCV coverage. We will use an adaptation of a method used to estimate indirect protection for an oral cholera vaccine which exploits heterogeneities in vaccine coverage at the subdistrict/village level, comparing VT carriage and density among under-vaccinated children from subdistricts/villages with differing levels of vaccine coverage.[33 34]

This will be done using multivariable models with VT carriage or density in ARI cases as the outcome variable and PCV coverage at the child's place of residence at the time of admission as the exposure variable. We will use generalised estimating equations to account for clustering at the subdistrict/village level.

To identify confounders for adjustment, we will constructed directed acyclic graphs (DAG). DAGs include all variables potentially related to exposure and outcome, connected using uni-directional arrows showing causal relationships between variables. The graph identifies potentially confounding pathways and allows investigators to determine variables that should be controlled for to obtain unbiased effect estimates. As there are likely to be unique confounders between sites, we will develop site-specific DAGs. We will use DAGitty.net (version 2.3) software to identify minimally sufficient confounding subsets for adjustment.

For each site, we will construct a similar model using overall pneumococcal carriage as the dependent variable. This model will act as a bias indicator since PCV coverage is not expected to affect levels of overall pneumococcal carriage due to replacement carriage with NVTs, although complete replacement to baseline levels can take several years.[21 35 36] Therefore, we will restrict this analysis to the latter part of the study period, when descriptive analyses indicate the replacement is complete.

To determine whether a higher PCV coverage is required to achieve indirect effects among completely un-vaccinated cases compared to under-vaccinated cases, we will conduct a sensitivity analysis among children who have never received PCV.

Objective 2: VT carriage prevalence among cases and contacts, by calendar month (PNG only)

Crude and adjusted monthly VT carriage prevalence will be estimated within rolling seven-month intervals to present smooth curves and assess trends over time. This will be done separately for cases and contacts. To account for differences in age between cases and community contacts, the carriage prevalence will be adjusted using direct standardisation (standardised to the case population over the entire study period).

Objective 3: Relationship between vaccine coverage and indirect effects among community contacts (PNG only)

To investigate whether relationship between vaccine coverage and indirect effects as observed among cases with ARI are reflective of the relationship between vaccine coverage and indirect effects in the wider community, we will apply the same model described above to under-vaccinated community contacts. We will construct multivariable models with VT carriage and density among

For peer review only - http://bmjopen.bmj.com/site/about/guidelines.xhtml

under-vaccinated community contacts as the outcome variable and PCV coverage at the child's place of residence at the time of admission as the exposure variable. We will be using the same vaccine coverage data as for the cases.

Objective 4: Comparison of vaccine coverage required for indirect effects across sites
We will compare differences in the PCV13 coverage required to demonstrate indirect effects of PCV13 qualitatively, by site and in relation to vaccine schedule and use of catch-up campaigns. Inferential statistics are unlikely to be suitable with the inclusion of only three sites and comparability between sites is limited due to variations between them.

Power calculation

Power calculations were performed using nQuery Advisor + nTerim 4.0. Calculations were based on sample size methods for logistic regression models with a continuous covariate (i.e. PCV coverage) and additional covariates, with inflation to account for clustering within villages. Power calculations assumed VT carriage prevalence of 30% in Lao PDR and 40% in Mongolia and PNG at the mean PCV coverage level,[37] and VT carriage prevalence of 20% in Lao PDR and 30% in Mongolia and PNG at one standard deviation above the mean PCV coverage level. Assuming a significance level of 0.05, allowing for adjustment using multiple covariates with an r-squared of 0.4, and that 50% of the cases are under-vaccinated, a sample size of 1200 cases per site would provide between 87% and 92% power to determine the proportion of cases carrying VT pneumococcus at varying levels of village vaccine coverage. The power calculation has been adjusted to account for clustering by village, with higher variability in Lao PDR and PNG (intraclass coefficient [ICC] 0.1) and lower variability in Mongolia (ICC 0.01).

Missing data

We will describe the number of participants with missing data on individual variables and compare the characteristics of those with and without missing data to determine whether there is evidence of systematic differences in characteristics. If we determine that the differences observed are able to be explained by available data (i.e. missing at random), we will consider using multiple imputation to predict the distribution of the missing data in order to account for the bias due to incomplete data.[38]

Patient and Public Involvement

Patients were not involved in the design of this study. Public health authorities in Laos and Mongolia were involved in the design and conduct of the study. In PNG, village representatives are approached to ensure that community surveys are conducted appropriately.

CURRENT STATUS OF THE STUDY

Recruitment is ongoing and analysis of data, followed by publication of results, is expected from 2018 onwards. As of August 2017, we have recruited 1039, 481 and 3847 cases from Lao PDR, PNG and Mongolia respectively. Table 4 describes the baseline characteristics of the children recruited.

For peer review only - http://bmjopen.bmj.com/site/about/guidelines.xhtml

Table 4: Case characteristics by site, Lao People's Democratic Republic (PDR), Mongolia and Papua New Guinea (PNG), 2014-2017

		Lao PDR<br>n=1039<br>n (%)	Mongolia<br>n=3847<br>n (%)	PNG<br>n=481<br>n (%)
Year of recruitment	2014	365/1039 (35)	NA	NA
	2015	323/1039 (31)	885/3847 (23)	NA
	2016	281/1039 (27)	2007/3847 (52)	190/481 (40)
	2017	70/1039 (7)	955/3847 (25)	291/481 (60)
Age group	< 12 months	432/1038 (42)	1481/3847 (39)	258/481 (54)
	12-23 months	346/1038 (33)	1250/3847 (32)	123/481 (25)
	>=24 months	260/1038 (25)	1116/3847 (29)	100/481 (21)
Gender	Male	591/1039 (57)	2079/3847 (54)	278/481 (58)

For peer review only

ETHICS AND DISSEMINATION

Prospective participants will be fully informed about the potential risks and benefits of participation and written informed consent will be obtained prior to recruitment. The study is being conducted according to protocols approved by the following ethics committees: Lao PDR Ministry of Health National Ethics Committee for Health Research (057/2013 NECHR), Oxford Tropical Research Ethics Committee (1050-13), Mongolian National Ethics Committee for Health Research, the WHO Regional Office for the Western Pacific (WPRO) Ethics Review Committee (2013.30.LAO.2.EPI), PNG IMR Institutional Review Board (1510), Government of PNG Medical Research Advisory Committee (15.18), and the Royal Children's Hospital/MCRI Human Research Ethics Committee (33177B, 33203E).

We plan on disseminating results to relevant stakeholders within Lao PDR, PNG and Mongolia, as well as submitting our findings for publication in relevant peer-reviewed journals and conferences.

DISCUSSION

The ability for low- and middle-income countries to monitor the indirect effects of PCV is critical. Maximising indirect effects is important because these effects comprise a substantial proportion of overall PCV impact and increase the cost-effectiveness of the vaccine.[39] Indirect protection is particularly important for individuals who are unable to be vaccinated or who have poor vaccine responses, such as infants too young to be vaccinated and the elderly. In addition, the Bill & Melinda Gates Foundation are supporting studies looking into the effectiveness of reduced dose schedules (1+1). This reduced dose schedule needs to maintain indirect effects following PCV introduction and this surveillance method provides a mechanism to determine when herd protection has been

For peer review only - http://bmjopen.bmj.com/site/about/guidelines.xhtml

achieved and whether it is maintained.[40] Furthermore, the ability to determine whether substantial herd immunity has been achieved may help to identify settings which are appropriate for introduction of reduced dose or modified schedules.

The results of our study will address the scarcity of literature determining the PCV coverage required to achieve substantial indirect effects, especially in low- and middle-income countries. The inclusion of three sites, which have contrasting vaccine schedules, baseline intensities of pneumococcal carriage and health-care systems, will also enable us to determine whether this vaccine coverage threshold differs by site and explore factors which may modify the vaccine coverage required to achieve indirect effects.

In this proposal, we describe a novel application of an analysis method to measure indirect effects. Although the isolation of a particular pneumococcal serotype from the nasopharynx of a child with ARI is not necessarily indicative of the serotype causing pneumococcal disease, reduced detection of VT pneumococci is likely to reflect reductions in disease due to VT pneumococci. Furthermore, our proposed analysis methods enable estimation of indirect effects in the absence of baseline pre-PCV data. This will be relevant for many low- and middle-income countries that have little or no baseline data and are considering options for surveillance to accompany the introduction of PCV.

Another key strength of our methods is the use of consistent molecular serotyping microarray methods across all three sites, enabling sensitive detection of multiple serotype carriage and ascertainment of serotype-specific density.[29] Conventional pneumococcal carriage studies typically detect the presence of a single serotype and may overlook the presence of vaccine serotypes occurring at lower densities. The results of this study will also add to the limited literature on the effects of PCV on vaccine-type carriage density, which may affect likelihood of transmission. [41]

Our proposed methods have several potential limitations. Firstly, there are significant challenges in determining accurate estimates of PCV coverage in resource-limited settings. In Mongolia and Lao PDR, where we are using administrative data, our estimates rely on the availability of accurate data about vaccine doses administered (numerator) as well as population estimates for the target age group (denominator). In both settings, we are conducting audits to assess the reliability of numerator data. Regarding denominator data, we are fortunate that a recent population census was conducted in Lao PDR in March 2015. However, we will be auditing denominator data in Mongolia, where population estimates are affected by large seasonal population movements between rural and urban settings. In PNG, the validity of the community surveys depends on representative sampling. However survey participation can vary and is affected by season (related to farming practices), as well as community trust and understanding of the study. To maximise participation, we conducted mobile health clinics alongside the community surveys.

The second main limitation relates to detecting pneumococcal carriage in our study population of children hospitalised with respiratory infection. In this population, carriage detection may be affected by the prior use of antibiotics.[42 43] To address this, we have, where possible, aimed to recruit patients

For peer review only - http://bmjopen.bmj.com/site/about/guidelines.xhtml

at admission, prior to receiving antibiotics or documented prior antibiotic use, which will be taken into account during analysis.

Thirdly, there are variations in methods and setting across the three sites. This limitation may impact on the comparability of our results across the three sites. The reason for the differences in methods between sites is that our study is built on pre-existing studies (pneumonia aetiology studies in Lao PDR and PNG, and a vaccine impact study in Mongolia) with established protocols.

To conclude, the results of this study will provide important feedback to national policy makers about the effects of newly introduced PCV programs in Lao PDR, Mongolia, and PNG. The results will help us understand the determinants of indirect effects and therefore guide strategies to maximise them. In particular, the results will also inform global policy about the vaccine coverage required to achieve substantial indirect effects in settings with different epidemiological characteristics of pneumococcal disease and carriage and will maximise the cost-effectiveness of the vaccine programs.

Acknowledgements:

We would like to acknowledge the Ministries of Health of Lao PDR and Mongolia, LOMWRU, PNG IMR and WHO and support from the Victorian Government's Operational Infrastructure Support Program. We would also like to thank study staff, laboratory staff and participating families.

The PneuCAPTIVE protocol development group includes the authors of the paper listed in the by-line and the following: Dashtseren Luvsantseren (NCCD, Ulaanbaatar, Mongolia), Bujinlkham (NCCD, Ulaanbaatar, Mongolia), Mukhchuluun Ulziibayar (NCCD, Ulaanbaatar, Mongolia), Dashpagam (NCCD, Ulaanbaatar, Mongolia), Audrey Dubot-Pérès (LOMHWRU, Vientiane, Lao PDR), Keodomphone Vilavong (LOMHWRU, Vientiane, Lao PDR), Anisone Chanthongthip (LOMHWRU, Vientiane, Lao PDR), Syladeth Chanthaphone (LOMHWRU, Vientiane, Lao PDR), Joycelyn Sapura (PNG IMR, Goroka, PNG), John Kave (PNG IMR, Goroka, PNG), Tonny Kumani (PNG IMR, Goroka, PNG), Wendy Kirarock (PNG IMR, Goroka, PNG).

Author's contributions: FR conceived the idea and designed the study. JL, SD, KF, PN, RL, RP, AX, DD and FR supported the development of country-specific protocols and study implementation in Lao PDR. CCB, RF, DL, WP and FR supported the development of country-specific protocols and study implementation in PNG. TM, SLV, CVM, KM, JC and FR supported the development of country-specific protocols and study implementation in Mongolia. CS, ED and JH devised the microbiological approach and laboratory protocols. JC, CN, RA, and FR devised the analysis plan. JC and FR drafted the manuscript. All authors provided feedback to the draft manuscript and have read and approved the final version.

Funding statement: This work is supported by the Bill & Melinda Gates Foundation grant number (OPP1115490). JC is completing a PhD at The University of Melbourne, funded by an Australian Government Research Training Program scholarship.

Competing interests: None declared.

For peer review only - http://bmjopen.bmj.com/site/about/guidelines.xhtml

REFERENCES

1. Wang H, Bhutta ZA, Coates MM, et al. Global, regional, national, and selected subnational levels of stillbirths, neonatal, infant, and under-5 mortality 1980-2015: a systematic analysis for the Global Burden of Disease Study 2015. *The Lancet* 2016;388(10053):1725-74. doi: 10.1016/S0140-6736(16)31575-6

2. Hill PC, Townend J, Antonio M, et al. Transmission of Streptococcus pneumoniae in rural Gambian villages: a longitudinal study. *Clinical Infectious Diseases* 2010;50(11):1468-76. doi: http://dx.doi.org/10.1086/652443

3. Hussain M, Melegaro A, Pebody RG, et al. A longitudinal household study of Streptococcus pneumoniae nasopharyngeal carriage in a UK setting. *Epidemiology and Infection* 2005;133(5):891-98. doi: http://dx.doi.org/10.1017/S0950268805004012

4. Gray BM, Converse GM, 3rd, Dillon HC, Jr. Epidemiologic studies of Streptococcus pneumoniae in infants: acquisition, carriage, and infection during the first 24 months of life. *The Journal of infectious diseases* 1980;142(6):923-33. [published Online First: 1980/12/01]

5. Varon E, Cohen R, Bechet S, et al. Invasive disease potential of pneumococci before and after the 13-valent pneumococcal conjugate vaccine implementation in children. *Vaccine* 2015;33(46):6178-85.

6. Johnson HL, Deloria-Knoll M, Levine OS, et al. Systematic evaluation of serotypes causing invasive pneumococcal disease among children under five: the pneumococcal global serotype project. *PLoS Med* 2010;7(10) doi: 10.1371/journal.pmed.1000348 [published Online First: 2010/10/20]

7. Loo JD, Conklin L, Fleming-Dutra KE, et al. Systematic review of the effect of pneumococcal conjugate vaccine dosing schedules on prevention of pneumonia. *The Pediatric infectious disease journal* 2014;33 Suppl 2:S140-51. doi: 10.1097/inf.0000000000000082 [published Online First: 2013/12/18]

8. Conklin L, Loo JD, Kirk J, et al. Systematic review of the effect of pneumococcal conjugate vaccine dosing schedules on vaccine-type invasive pneumococcal disease among young children. *The Pediatric infectious disease journal* 2014;33 Suppl 2:S109-18. doi: 10.1097/inf.0000000000000078 [published Online First: 2013/12/18]

9. Le Saux N. Pneumococcal conjugate vaccines for preventing otitis media. *Paediatrics & Child Health* 2016;21(2):89-90.

10. Loo JD, Conklin L, Fleming-Dutra KE, et al. Systematic review of the indirect effect of pneumococcal conjugate vaccine dosing schedules on pneumococcal disease and colonization. *Pediatric Infectious Disease Journal* 2014;33 Suppl 2:S161-71.

11. Direct and indirect effects of routine vaccination of children with 7-valent pneumococcal conjugate vaccine on incidence of invasive pneumococcal disease--United States, 1998-2003. *MMWR Morbidity and mortality weekly report* 2005;54(36):893-7. [published Online First: 2005/09/16]

12. Tsaban G, Ben-Shimol S. Indirect (herd) protection, following pneumococcal conjugated vaccines introduction: A systematic review of the literature. *Vaccine* 2017;35(22):2882-91. doi: 10.1016/j.vaccine.2017.04.032 [published Online First: 2017/04/30]

13. Loughlin AM, Hsu K, Silverio AL, et al. Direct and indirect effects of PCV13 on nasopharyngeal carriage of PCV13 unique pneumococcal serotypes in Massachusetts' children. *The Pediatric infectious disease journal* 2014;33(5):504-10. doi: 10.1097/inf.0000000000000279 [published Online First: 2014/03/29]

14. Grant LR, Hammitt LL, O'Brien SE, et al. Impact of the 13-valent pneumococcal conjugate vaccine on pneumococcal carriage among American Indians. *The Pediatric infectious disease journal* 2016;35(8):907-14. doi: 10.1097/inf.0000000000001207 [published Online First: 2016/05/14]

For peer review only - http://bmjopen.bmj.com/site/about/guidelines.xhtml

15. International Vaccine Access Center - John Hopkins Bloomberg School of Public Health. VIEW-hub 2016 [accessed 17/12/2016.

16. Shiri T, Datta S, Madan J, et al. Indirect effects of childhood pneumococcal conjugate vaccination on invasive pneumococcal disease: a systematic review and meta-analysis. *The Lancet Global Health* 2017;5(1):e51-e59. doi: 10.1016/s2214-109x(16)30306-0

17. Jayasinghe S, Menzies R, Chiu C, et al. Long-term impact of a "3+0" schedule for 7 and 13 valent pneumococcal conjugate vaccines on invasive pneumococcal disease in Australia, 2002-2014. *Clinical Infectious Diseases* 2016 doi: 10.1093/cid/ciw720

18. Mackenzie GA, Hill PC, Jeffries DJ, et al. Effect of the introduction of pneumococcal conjugate vaccination on invasive pneumococcal disease in The Gambia: a population-based surveillance study. *The Lancet Infectious diseases* 2016;16(6):703-11. doi: 10.1016/s1473-3099(16)00054-2 [published Online First: 2016/02/22]

19. von Gottberg A, de Gouveia L, Tempia S, et al. Effects of vaccination on invasive pneumococcal disease in South Africa. *The New England journal of medicine* 2014;371(20):1889-99. doi: 10.1056/NEJMoa1401914 [published Online First: 2014/11/12]

20. Rodgers GL, Klugman KP. Surveillance of the impact of pneumococcal conjugate vaccines in developing countries. *Human Vaccines and Immunotherapeutics* 2016;12(2):417-20. doi: http://dx.doi.org/10.1080/21645515.2015.1057671

21. Gladstone RA, Jefferies JM, Tocheva AS, et al. Five winters of pneumococcal serotype replacement in UK carriage following PCV introduction. *Vaccine* 2015;33(17):2015-21.

22. Dunne EM, Manning J, Russell FM, et al. Effect of pneumococcal vaccination on nasopharyngeal carriage of Streptococcus pneumoniae, Haemophilus influenzae, Moraxella catarrhalis, and Staphylococcus aureus in Fijian children. *J Clin Microbiol* 2012;50(3):1034-8. doi: 10.1128/jcm.06589-11 [published Online First: 2011/12/16]

23. Mosser JF, Grant LR, Millar EV, et al. Nasopharyngeal carriage and transmission of Streptococcus pneumoniae in American Indian households after a decade of pneumococcal conjugate vaccine use.[Erratum appears in PLoS One. 2014;9(3):e93878]. *PLoS ONE [Electronic Resource]* 2014;9(1):e79578.

24. Lehmann D, Gratten M, Montgomery J. Susceptibility of pneumococcal carriage isolates to penicillin provides a conservative estimate of susceptibility of invasive pneumococci. *The Pediatric infectious disease journal* 1997;16(3):297-305. [published Online First: 1997/03/01]

25. Nguyen VH, Dubot-Peres A, Russell FM, et al. Acute respiratory infections in hospitalized children in Vientiane, Lao PDR - the importance of Respiratory Syncytial Virus. *Scientific reports* 2017;7(1):9318. doi: 10.1038/s41598-017-09006-6 [published Online First: 2017/08/26]

26. Blyth CC, Ford R, Sapura J, et al. Childhood pneumonia and meningitis in the Eastern Highlands Province, Papua New Guinea in the era of conjugate vaccines: study methods and challenges. *Pneumonia (Nathan Qld)* 2017;9:5. doi: 10.1186/s41479-017-0029-y [published Online First: 2017/07/14]

27. Satzke C, Turner P, Virolainen-Julkunen A, et al. Standard method for detecting upper respiratory carriage of Streptococcus pneumoniae: updated recommendations from the World Health Organization Pneumococcal Carriage Working Group. *Vaccine* 2013;32(1):165-79. doi: 10.1016/j.vaccine.2013.08.062 [published Online First: 2013/12/18]

28. Dunne EM, Manning J, Russell FM, et al. Effect of Pneumococcal Vaccination on Nasopharyngeal Carriage of Streptococcus pneumoniae, Haemophilus influenzae, Moraxella catarrhalis, and Staphylococcus aureus in Fijian Children. *Journal of Clinical Microbiology* 2012;50(3):1034-38. doi: 10.1128/JCM.06589-11

For peer review only - http://bmjopen.bmj.com/site/about/guidelines.xhtml

29. Satzke C, Dunne EM, Porter BD, et al. The PneuCarriage Project: A Multi-Centre Comparative Study to Identify the Best Serotyping Methods for Examining Pneumococcal Carriage in Vaccine Evaluation Studies. *PLoS Medicine / Public Library of Science* 2015;12(11):e1001903; discussion e03.

30. Newton R, Hinds J, Wernisch L. Empirical Bayesian models for analysing molecular serotyping microarrays. *BMC bioinformatics* 2011;12:88. doi: 10.1186/1471-2105-12-88 [published Online First: 2011/04/02]

31. Scott P, Rutjes AW, Bermetz L, et al. Comparing pneumococcal conjugate vaccine schedules based on 3 and 2 primary doses: systematic review and meta-analysis. *Vaccine* 2011;29(52):9711-21. doi: 10.1016/j.vaccine.2011.07.042 [published Online First: 2011/08/09]

32. Chan J. High agreement between the new Mongolian electronic immunisation register and clinic immunisation records: a health centre based audit. *Western Pacific Surveillance and Response Journal* 2017;In press

33. Deen J, Ali M, Sack D. Methods to assess the impact of mass oral cholera vaccination campaigns under real field conditions. *PLoS ONE* 2014;9(2):e88139. doi: 10.1371/journal.pone.0088139

34. Khatib AM, Ali M, von Seidlein L, et al. Effectiveness of an oral cholera vaccine in Zanzibar: findings from a mass vaccination campaign and observational cohort study. *The Lancet Infectious diseases* 2012;12(11):837-44. doi: 10.1016/s1473-3099(12)70196-2 [published Online First: 2012/09/08]

35. Moore MR, Hyde TB, Hennessy TW, et al. Impact of a conjugate vaccine on community-wide carriage of nonsusceptible Streptococcus pneumoniae in Alaska. *Journal of Infectious Diseases* 2004;190(11):2031-8.

36. Usuf E, Bottomley C, Adegbola RA, et al. Pneumococcal carriage in sub-Saharan Africa - A systematic review. *PLoS ONE* 2014;9 (1) (no pagination)(e85001) doi: http://dx.doi.org/10.1371/journal.pone.0085001

37. Aho C, Michael A, Yoannes M, et al. Limited impact of neonatal or early infant schedules of 7-valent pneumococcal conjugate vaccination on nasopharyngeal carriage of Streptococcus pneumoniae in Papua New Guinean children: A randomized controlled trial. *Vaccine Reports* 2016;6:36-43. doi: http://dx.doi.org/10.1016/j.vacrep.2016.08.002

38. Sterne JAC, White IR, Carlin JB, et al. Multiple imputation for missing data in epidemiological and clinical research: potential and pitfalls. *Bmj* 2009;338 doi: 10.1136/bmj.b2393

39. Isaacman DJ, Strutton DR, Kalpas EA, et al. The impact of indirect (herd) protection on the cost-effectiveness of pneumococcal conjugate vaccine. *Clinical therapeutics* 2008;30(2):341-57. doi: 10.1016/j.clinthera.2008.02.003 [published Online First: 2008/03/18]

40. Rodgers GL, Klugman KP. A new paradigm in pneumococcal conjugate vaccination: moving from individual to herd protection. *International journal of infectious diseases : IJID : official publication of the International Society for Infectious Diseases* 2017 doi: 10.1016/j.ijid.2017.04.015

41. Roca A, Bottomley C, Hill PC, et al. Effect of age and vaccination with a pneumococcal conjugate vaccine on the density of pneumococcal nasopharyngeal carriage. *Clinical Infectious Diseases* 2012;55(6):816-24.

42. Abdullahi O, Karani A, Tigoi CC, et al. The prevalence and risk factors for pneumococcal colonization of the nasopharynx among children in Kilifi District, Kenya. *PLoS One* 2012;7(2):e30787. doi: 10.1371/journal.pone.0030787 [published Online First: 2012/03/01]

43. Alpkvist H, Athlin S, Naucler P, et al. Clinical and Microbiological Factors Associated with High Nasopharyngeal Pneumococcal Density in Patients with Pneumococcal Pneumonia. *PLoS One* 2015;10(10):e0140112. doi: 10.1371/journal.pone.0140112 [published Online First: 2015/10/16]

For peer review only - http://bmjopen.bmj.com/site/about/guidelines.xhtml

For peer review only

For peer review only - http://bmjopen.bmj.com/site/about/guidelines.xhtml
